# $C^\infty$ regularity in semilinear free boundary problems

**DOI:** 10.1007/s00208-025-03135-4

**Published:** 2025-05-07

**Authors:** Daniel Restrepo, Xavier Ros-Oton

**Affiliations:** 1 Department of Mathematics, Johns Hopkins University, 3400 N. Charles Street, Baltimore, MD 21218 USA; 2 ICREA, Pg. Lluís Companys 23, 08010 Barcelona, Spain; 3 Departament de Matemàtiques i Informàtica, Universitat de Barcelona, Gran Via de les Corts Catalanes 585, 08007 Barcelona, Spain; 4 Centre de Recerca Matemàtica, Barcelona, Spain

**Keywords:** 35R35, 35B65, 35J61, 35J25

## Abstract

We study the higher regularity of solutions and free boundaries in the Alt–Phillips problem $\Delta u=u^{\gamma -1},$ with $\gamma \in (0,1).$ Our main results imply that, once free boundaries are $C^{1,\alpha },$ then they are $C^\infty .$ In addition $u/d^{\frac{2}{2-\gamma }}$ and $u^{\frac{2-\gamma }{2}}$ are $C^\infty$ too. In order to achieve this, we need to establish fine regularity estimates for solutions of linear equations with boundary-singular Hardy potentials $-\Delta v = \kappa v/d^2$ in $\Omega ,$ where *d* is the distance to the boundary and $\kappa \le \frac{1}{4}.$ Interestingly, we need to include even the critical constant $\kappa =\frac{1}{4},$ which corresponds to $\gamma =\frac{2}{3}.$

## Introduction

A classical problem in the Calculus of Variations concerns the study of minimizers of functionals of the type

$$\begin{aligned} J(u):= \int _{D} {\textstyle \frac{1}{2}}|\nabla u|^2 +F(u), \end{aligned}$$

with boundary data $u=g\ge 0$ on $\partial D.$ The potential *F* is usually asked to be nondecreasing, and with $F(0)=0.$ Under these conditions, minimizers of *J* are non-negative and satisfy (in an appropriate sense) the Euler–Lagrange equation

1.1 $$\begin{aligned} \Delta u = F'(u)\quad \text{ in }\ D. \end{aligned}$$

Two of the most famous and important cases are $F(u)=u_+$ (the obstacle problem), and $F(u)=\chi _{\{u>0\}}$ (the Alt–Caffarelli or Bernoulli free boundary problem). In general, when $F\notin C^{1,1},$ the solution will develop free boundaries: there will be a region of *D* in which $u=0,$ and a region in which $u>0.$ The so-called free boundary is the interface $\partial \{u>0\}.$

In this paper we are interested in the more general family of problems

$$\begin{aligned} F(u)={\textstyle \frac{1}{\gamma }}u^\gamma \quad \text{ with }\ \gamma \in (0,2), \end{aligned}$$

which gives rise to the so-called Alt–Phillips problem, introduced in [4]. This family of problems obviously encompasses both the obstacle problem $(\gamma =1),$ as well as the Alt–Caffarelli problem $(\gamma \rightarrow 0).$

The regularity theory for both the obstacle and the Alt–Caffarelli problems is quite well understood, and in particular the free boundary is known to be $C^\infty$ outside a closed set of singular points [10, 17, 30, 34]. Moreover, fine properties of the singular set have been established in [16, 20, 22, 25].

The regularity theory for minimizers in the Alt–Phillips problem has been developed in the works [4, 6, 15, 19, 36], and the main known results may be summarized as follows:

1. Solutions *u* are $C^{\frac{2}{2-\gamma }},$ and this is optimal.^1^
2. Free boundary points can be split into two sets: regular points, and singular points.
3. The regular set is an open subset of the free boundary, and it is $C^{1,\alpha }.$
4. The singular set is closed, and:
  When $\gamma \in (0,1)$ its Hausdorff dimension is at most $n-3.$
  When $\gamma \in [1,2)$ it is $(n-1)$-rectifiable.

Our main goal in this paper is to prove that the set of regular points is actually $C^\infty ,$ and that solutions enjoy a $C^\infty$ regularity estimate near regular points, namely $u/d^{\frac{2}{2-\gamma }}\in C^\infty .$

This has been only proved for $\gamma \in (1,2)$ by Fotouhi–Koch [21] and very recently for $\gamma \in (\frac{2}{3},2)$ by Allen–Kriventsov–Shahgholian [2], and has been open for a very long time in the case $\gamma \in (0,\frac{2}{3}].$ Our proof is completely different from that in [2, 21], and covers the whole range of exponents $\gamma \in (0,2).$

As we will see, quite interestingly, we find that in terms of higher order regularity the problem behaves like the obstacle problem for $\gamma \ge \frac{2}{3},$ while it is more similar to the Bernoulli problem for $\gamma <\frac{2}{3}.$ This is quite new and surprising, since in almost all previous regularity results the only differences were in cases $\gamma \in (1,2)$ vs $\gamma \in (0,1).$

### Main result

Our main result can be stated as follows. Recall that regular free boundary points are those at which the free boundary $\partial \{u>0\}$ is $C^{1,\alpha }$ in a neighborhood.

#### Theorem 1.1

Let $\gamma \in (0,2)$ and $0\le u \in C^{\frac{2}{2-\gamma }}$ be any viscosity solution of

1.2 $$\begin{aligned} \Delta u = u^{\gamma -1} \quad \text {in}\ \{u>0\}\cap B_1. \end{aligned}$$

Assume $x_0 \in \partial \{u>0\}\cap B_1$ is a regular free boundary point. Then, 

- The free boundary $\partial \{u>0\}$ is $C^\infty$ in a neighborhood of $x_0.$
- The functions $u/d^{\frac{2}{2-\gamma }}$ and $u^{\frac{2-\gamma }{2}}$ are $C^\infty$ in a neighborhood of $x_0.$

In addition to its own intrinsic interest, this result is important for example in the study of stable solutions to the Alt–Phillips problem (in which one needs at least second order expansions for solutions near $\partial \Omega ),$ especially when $\gamma < \frac{2}{3};$ see [26].

Our approach to prove Theorem 1.1 is to find a PDE for the derivatives $$u_i$$ (and for their quotients $$u_i/u_j),$$ to then use these equations to deduce that solutions and free boundaries are smooth. This approach was introduced by De Silva and Savin in the context of the obstacle problem [13], and also in the Alt–Caffarelli problem; see [14].

In the obstacle problem, they proved that if $v_2>0$ in $\Omega \cap B_1,$ then

$$\begin{aligned} \left. \begin{array}{r} \Delta v_1=\Delta v_2=0 \quad \text {in}\ \Omega \cap B_1\\ v_1=v_2=0 \quad \text {on}\ \partial \Omega \cap B_1\\ \partial \Omega \quad \text {is}\ C^{k,\alpha }\\ \end{array}\right\} \qquad \Longrightarrow \qquad \frac{v_1}{v_2} \in C^{k,\alpha }({{\overline{\Omega }}} \cap B_{1/2}), \end{aligned}$$

for any $k\ge 1$ and $\alpha \in (0,1).$ This, applied to the derivatives $u_i,$ and using that the normal vector $\nu$ to $\partial \Omega$ can be written as

$$\begin{aligned} \nu ^i = \frac{u_i}{|\nabla u|} = \frac{u_i/u_n}{\sqrt{\sum _j (u_j/u_n)^2}} \end{aligned}$$

yields that

1.3 $$\begin{aligned} \partial \Omega \in C^{k,\alpha } \quad \Longrightarrow \quad \frac{u_i}{u_n}\in C^{k,\alpha } \quad \Longrightarrow \quad \nu \in C^{k,\alpha } \quad \Longrightarrow \quad \partial \Omega \in C^{k+1,\alpha }, \end{aligned}$$

thus yielding the $C^\infty$ regularity by a bootstrap argument.

In case of the Alt–Caffarelli free boundary problem, the argument goes in the same spirit, but the PDE is completely different. It turns out that the quotient $w:=u_i/u_n$ solves

$$\begin{aligned}\begin{aligned} {\textrm{div}}(u_n^2 \nabla w)=0&\quad \text {in}\ \Omega \cap B_1 \\ \partial _\nu w =0&\quad \text {on}\ \partial \Omega \cap B_1, \end{aligned}\end{aligned}$$

and thus by Schauder estimates we get

1.4 $$\begin{aligned} \partial \Omega \in C^{k,\alpha } \quad \Longrightarrow \quad u_n\in C^{k-1,\alpha } \quad \Longrightarrow \quad w\in C^{k,\alpha } \Longrightarrow \quad \nu \in C^{k,\alpha } \quad \Longrightarrow \quad \partial \Omega \in C^{k+1,\alpha }.\nonumber \\ \end{aligned}$$

This yields that $\partial \Omega$ is $C^\infty ,$ and thus applying now Schauder estimates (with Dirichlet conditions) to the solution *u*,  we find that $u\in C^\infty .$

### The linearized equation

In our setting, derivatives $u_i$ are not harmonic but solve the linearized equation

$$\begin{aligned} \Delta u_i = (\gamma -1)u^{\gamma -2} u_i \quad \text {in}\ \Omega \cap B_1. \end{aligned}$$

At regular points, we know that

$$\begin{aligned} u \sim c_\gamma d^{\frac{2}{2-\gamma }} \end{aligned}$$

where *d* is the distance to the (free) boundary and^2^

1.5 $$\begin{aligned} c_\gamma := \left( \frac{(2-\gamma )^2}{2 \gamma }\right) ^\frac{1}{2-\gamma }; \end{aligned}$$

see [4]. Thus, since near regular points we also know that $u/d^{\frac{2}{2-\gamma }} \in C^\alpha ,$ then the linearized equation becomes

1.6 $$\begin{aligned} \Delta u_i = \kappa _\gamma \frac{u_i}{d^2} + f d^{\frac{\gamma }{2-\gamma }-2} \quad \text {in}\ \Omega \cap B_1, \end{aligned}$$

where $$f|_{\partial \Omega }=0,$$ $$f\in C^\alpha ({{\overline{\Omega }}}\cap B_1),$$ and

1.7 $$\begin{aligned} \kappa _\gamma := (\gamma -1)c_\gamma ^{\gamma -2}= \Big (\frac{\gamma }{2-\gamma }\Big ) \Big (\frac{\gamma }{2-\gamma }-1\Big ). \end{aligned}$$

Notice that

$$\begin{aligned} \kappa _\gamma \ge -{\textstyle \frac{1}{4}}, \end{aligned}$$

with the minimum being attained at $\gamma =\frac{2}{3}.$ Moreover, we also have the symmetry property

$$\begin{aligned} \kappa _\gamma = \kappa _{\frac{4-4\gamma }{4-3\gamma }}. \end{aligned}$$

Thus, we define the linearized operator

1.8 $$\begin{aligned} L_\kappa (v):=-\Delta v + \kappa \,\frac{v}{d^2}. \end{aligned}$$

Quite interestingly, this operator is the one appearing in the Euler–Lagrange equation of the Hardy-type inequality

$$\begin{aligned} \int _\Omega |\nabla \xi |^2 + \kappa \int _\Omega \frac{\xi ^2}{d^2} \ge 0, \end{aligned}$$

which holds in bounded smooth domains for $\kappa >-\frac{1}{4},$ and *does not* hold for $\kappa <-\frac{1}{4}.$ In case $\kappa = - \frac{1}{4}$ the inequality is known to hold in convex domains. While we will not use such variational characterization, this shows that the range $\kappa \ge -\frac{1}{4}$ is very natural, and the case $\kappa =- \frac{1}{4}$ is critical.

Our analysis will be based on non-variational arguments, and in particular we will establish a comparison principle for $L_\kappa$ as well as suitable barriers.

As we will see, there are two characteristic powers for the operator in dimension one:

$$\begin{aligned} L_\kappa (x^\theta ) = 0 \quad \text {for}\ x>0 \qquad \Longleftrightarrow \qquad \theta =\frac{1\pm \sqrt{1+4\kappa }}{2}. \end{aligned}$$

We call the largest root $\theta _+,$ whereas we will denote by $\theta _-$ the smallest root. The first one will behave somehow as the exponent of solutions with Dirichlet conditions, while the second one somehow corresponds to the exponent of solutions with Neumann conditions. The case $\kappa =- \frac{1}{4}$ is slightly different, as there is only one exponent $x^{1/2},$ but then there is a second 1D solution, namely $x^{1/2}\log x.$

Our main result for the linearized operator $L_\kappa ,$ which we believe is of independent interest, reads as follows.

#### Theorem 1.2

Let $\kappa \in [-\frac{1}{4},\infty ),$ and let $\Omega \subset \mathbb {R}^n$ be a $C^{k+1,\alpha }$ domain,  $k\ge 0$ and $\alpha \in (0,1).$ Let $f\in C^{k,\alpha }( {{\overline{\Omega }}} \cap B_1 )$ be such that $f|_{\partial \Omega }\equiv 0,$ and *d* be the regularized distance to $\partial \Omega ,$ given by Lemma  2.1.

Let $v \in C(\Omega \cap B_1)$ be any solution to

1.9 $$\begin{aligned} -\Delta v + \kappa \,\frac{v}{d^2}=fd^{\theta _+-2}\quad \text {in}\ \Omega \cap B_1 , \end{aligned}$$

satisfying

1.10 $$\begin{aligned} \begin{array}{rl} \displaystyle \lim _{x\rightarrow \partial \Omega } \frac{v}{d^{\theta _-}} = 0 &  \quad \text {if}\ \kappa >-{\textstyle \frac{1}{4}} \\ \displaystyle \lim _{x\rightarrow \partial \Omega } \frac{v}{d^{1/2} |\log d|} = 0 &  \quad \text {if}\ \kappa =-{\textstyle \frac{1}{4}} . \end{array} \end{aligned}$$

Then,  $$v\in C^{\theta _+}( B_{1/2}\cap {{\overline{\Omega }}})$$ and

$$\begin{aligned} \big \Vert v/{d^{\theta _+}}\big \Vert _{C^{k,\alpha }({{\overline{\Omega }}} \cap B_{1/2})}\le C\big (\Vert f\Vert _{C^{k,\alpha }({{\overline{\Omega }}}\cap B_1)} + \Vert v\Vert _{L^\infty (\Omega \cap B_1)} \big ), \end{aligned}$$

where $C>0$ depends only on $\Omega ,$ *k*,  $\alpha ,$ *n*,  and $\kappa .$

Here, the assumption (1.10) acts as a Dirichlet boundary condition on $\partial \Omega$ ruling out the existence of solutions behaving like the other possible power. This is interesting because *v* is not assumed to be bounded and since $\theta _- <0$ when $\kappa >0,$ (1.10) imposes a growth bound nearby the boundary which, a posteriori, implies the boundedness of *v*.

We will also prove a modified version of the result allowing for general pointwise expansions for *v* of the form $Q_1 d^{\theta _+}+ Q_2 d^{\theta _-+1}$ nearby $\partial \Omega$ with $Q_1$ and $Q_2$ polynomials of a suitable degree; see Theorem 4.2.

### Strategy of the proofs

Recall that solutions *u* to the Alt–Phillips problem behave like $d^{\frac{2}{2-\gamma }},$ and thus their derivatives $u_i$ do so like $d^{\frac{\gamma }{2-\gamma }}.$ This means that we have two completely different cases: for $\gamma \ge \frac{2}{3}$ the exponent $\frac{\gamma }{2-\gamma }$ corresponds to $\theta _+$ of the linearized operator $L_\kappa$

$$\begin{aligned} \gamma \in [{\textstyle \frac{2}{3}},2) \qquad \Longrightarrow \qquad u_i \asymp d^{\theta _+}, \end{aligned}$$

while for $\gamma <\frac{2}{3}$ the exponent $\frac{\gamma }{2-\gamma }$ corresponds to $\theta _-$ of $L_\kappa$

$$\begin{aligned} \gamma \in (0,{\textstyle \frac{2}{3}}) \qquad \Longrightarrow \qquad u_i \asymp d^{\theta _-}. \end{aligned}$$

In a sense, this means that *when* $\gamma \ge \frac{2}{3}$ *derivatives* $u_i$ *have zero Dirichlet boundary data*, while for $\gamma <\frac{2}{3}$ this is not true anymore.

In both cases, the idea is to apply Theorem 1.2 inductively to show that $u_i/d^{\frac{\gamma }{2-\gamma }},$ $u/d^{\frac{2}{2-\gamma }},$ and $u_i/u_n$ are $C^\infty ,$ namely,

1.11 $$\begin{aligned} \partial \Omega \in C^{k+1,\alpha } \quad \overset{(a)}{\Longrightarrow }\ \quad \frac{u_i}{d^{\frac{\gamma }{2-\gamma }}}, \frac{u}{d^{\frac{2}{2-\gamma }}}\in C^{k,\alpha } \quad \overset{(b)}{\Longrightarrow } \quad \frac{u_i}{u_n} \in C^{k+1,\alpha } \quad \Longrightarrow \quad \partial \Omega \in C^{k+2,\alpha }.\nonumber \\ \end{aligned}$$

Let us notice that since nearby regular points $u/d^{\frac{2}{2-\gamma }} \in C^\alpha$ (see Lemma 2.3), from the scheme (1.11), (a) only has to be considered when $k\ge 1.$ However, it is not immediate to apply Theorem 1.2 to the derivatives $u_i$ in order to get (a). Indeed, by induction hypothesis we know that $u/d^{\frac{2}{2-\gamma }}\in C^{k-1,\alpha }$ and $u_i/d^{\frac{\gamma }{2-\gamma }}\in C^{k-1,\alpha },$ so that the right hand side *f* in the Eq. (1.6) is then $C^{k-1,\alpha }.$ Unfortunately, using Theorem 1.2 (when $\gamma \ge \frac{2}{3})$ we then deduce that $u_i/d^{\frac{\gamma }{2-\gamma }}\in C^{k-1,\alpha },$ gaining no new information.

To improve the smoothness of $u/d^{\frac{\gamma }{2-\gamma }}$ and $u_i/d^{\frac{\gamma }{2-\gamma }},$ a key idea is to consider the function

$$\begin{aligned} v:=u-c_\gamma d^{\frac{2}{2-\gamma }}, \end{aligned}$$

and use the identity

$$\begin{aligned}  &   p^{\gamma -1}-q^{\gamma -1}=(\gamma -1)(p-q)q^{\gamma -2} + (p/q-1)^2 q^{\gamma -1}\\  &   \quad \int _0^1 (\gamma -1)(\gamma -2) \big (1+t p/q\big )^{\gamma -3}(1-t) dt \end{aligned}$$

for $p,q>0,$ to show that

1.12 $$\begin{aligned} L_\kappa v= &   u^{\gamma -1}-\big (c_\gamma d^{\frac{2}{2-\gamma }}\big )^{\gamma -1}+gd^{\frac{2}{2-\gamma }-2} \nonumber \\= &   \kappa \frac{v}{d^2} + f d^{\frac{2}{2-\gamma }-2} + gd^{\frac{2}{2-\gamma }-2}, \end{aligned}$$

where $$g\in C^{k,\alpha }$$ and

$$\begin{aligned} f:= \big (u/d^{\frac{2}{2-\gamma }}-c_\gamma \big )^2 \int _0^1 \bar{c}_\gamma \big (c_\gamma +t u/d^{\frac{2}{2-\gamma }}\big )^{\gamma -3}(1-t) dt.\end{aligned}$$

The crucial point here is that, even though by inductive hypothesis we only know $u/d^{\frac{2}{2-\gamma }}$ is $C^{k-1,\alpha },$ we will show that *f* is actually *more* regular, namely $f\in C^{k,\alpha }$ if $k\ge 2.$

The reason for this is that, roughly speaking, if a function *w* is *pointwise* $C^{m,\alpha }$ at $x=0,$ and $w(0)=0,$ then $w^2$ is actually pointwise $C^{m+1,\alpha }$ at $x=0$ if^3^$m\ge 1.$ And $w^2\eta$ will still be pointwise $C^{m+1,\alpha }$ as long as $\eta$ is $C^{m,\alpha }.$ This, yields that *f* is pointwise $C^{k,\alpha }$ at all boundary points, and combined with interior estimates for *u* allows us to prove that *f* is indeed $C^{k,\alpha }$ in ${{\overline{\Omega }}}.$ Combining this with the fact that *v* satisfies simultaneously (1.10) and (4.47), it is possible to prove a modified version of Theorem 1.2, i.e., Theorem 5.1, to deduce $v/d^{\frac{2}{2-\gamma }} \in C^{k,\alpha }.$

Recalling the definition of *v*,  and using again interior estimates for *u*,  we then prove the desired estimate $u_i/d^{\frac{\gamma }{2-\gamma }} \in C^{k,\alpha }$ in (a).

Once we have (a) in (1.11), to prove (b) we notice that the quotient $w:=u_i/u_n$ satisfies

$$\begin{aligned}\begin{aligned} {\textrm{div}}(u_n^2 \nabla w)=0&\quad \text {in}\ \Omega \cap B_1 \\ \partial _\nu w =0&\quad \text {on}\ \partial \Omega \cap B_1, \end{aligned}\end{aligned}$$

which is a degenerate elliptic equation since $u_n\rightarrow 0$ on $\partial \Omega .$ Still, thanks to (a), we have $u_n^2=d^s a(x),$ with $s=\frac{2\gamma }{2-\gamma }$ and $a(x)\in C^{k,\alpha }.$ Using this, we can apply^4^ Schauder estimates for degenerate elliptic equations with Neumann boundary conditions due to Terraccini–Tortone–Vita [32], to show that $w\in C^{k+1,\alpha }.$

Although a key point in showing (a)—for all values of $\gamma \in (0,2)$—is Theorem 5.1 instead of Theorem 1.2, the former is actually a consequence of the latter. Also, Theorem 1.2 has direct applications to the study of the boundary behavior of solutions to equations involving operators with Hardy-type potentials (see [24]).

To prove Theorem 1.2, we first need a comparison principle for the operator $L_\kappa$ with boundary conditions (1.10). We prove it by constructing explicit barriers, and reads as follows:

#### Lemma 1.3

Let $\kappa \in [-\frac{1}{4},\infty ),$ $\alpha \in (0,1),$ $\Omega \subset \mathbb {R}^n$ be such that $\Omega \cap B_1$ is a $C^{1,\alpha }$ domain,  and *d* be as in Theorem 1.2.

Let $v \in C( \Omega \cap B_1)$ be any viscosity solution of

$$\begin{aligned} -\Delta v + \kappa \,\frac{v}{d^2}\le 0\quad \text {in}\ \Omega \cap B_1 \end{aligned}$$

satisfying

1.13 $$\begin{aligned} \begin{array}{rl} \displaystyle \lim _{x\rightarrow \partial \Omega } \frac{v}{d^{\theta _-}} \le 0 &  \quad \text {if}\ \ \kappa >-{\textstyle \frac{1}{4}}\\ \displaystyle \lim _{x\rightarrow \partial \Omega } \frac{v}{d^{1/2} |\log d|} \le 0 &  \quad \text {if}\ \ \kappa =-{\textstyle \frac{1}{4}} . \end{array} \end{aligned}$$

Then,  there exists $\rho \in (0,1)$ only depending on the $C^{1,\alpha }$ norm of $\partial \Omega$ such that for any $r\in (0,\rho ]$ if $v\le 0$ on $\partial \Omega \cap B_r,$ then $v\le 0$ in $\Omega \cap B_r.$

Using this, we prove uniform $C^{\theta _+}$ estimates for solutions to the linearized equation $L_\kappa v=0$ with zero Dirichlet boundary conditions. This gives us compactness on the family of solutions *v* we are studying, and thus we can run a contradiction and blow-up argument to get the higher order expansion, which is developed in Sect. 4. This, combined with a classification of global solutions in a half-space (see Proposition 3.5), will yield Theorem 1.2, as well as its generalized version Theorem 4.2.

### Organization of the paper

In Sect. 2 we give some preliminary lemmas. Then, in Sects. 3 and 4 we develop the boundary regularity for the linearized operator $L_\kappa ,$ proving in particular Theorem 1.2. Finally, in Sect. 5 we prove Theorem 1.1, while in Sect. 6 we give some technical lemmas that are used throughout the proofs.

### Notations and conventions

We denote ${\textbf{P}}_{k}$ the space of polynomials of order *k* in *n* variables. Polynomials will be written in their coefficient expansion following convention

$$\begin{aligned} Q \in {\textbf{P}}_{k} \qquad \implies \qquad Q(x)= \sum _{\alpha \in \mathbb {N}^n,\, |\alpha |\le k} q^{(\alpha )}x^\alpha , \quad x\in \mathbb {R}^n. \end{aligned}$$

In the study of boundary regularity in a domain $\Omega ,$ we will always assume that $0\in \partial \Omega$ and, whenever $\Omega$ is regular at 0,  we will orient it so that the canonical vector $-e_n$ corresponds to its outer unit normal at zero, unless otherwise stated. Note that these two simplifications are possible thanks to the translation and rotation invariances of (1.2). In concordance with this assumption, we will adopt the notation $x= (x',x_n) \in \mathbb {R}^{n-1}\times \mathbb {R},$ calling the first $n-1$ coordinates “tangential”, whereas we will refer to the coordinate $x_n$ as the normal direction.

Associated to $\kappa \in [\frac{-1}{4}, \infty ),$ we define the quantities

1.14 $$\begin{aligned} \theta _+ =\frac{1+\sqrt{1+4\kappa }}{2}, \qquad \theta _-= \frac{1-\sqrt{1+4\kappa }}{2}, \end{aligned}$$

which arise in the study of the linearized operator (1.8) as discussed in the introduction (see also Lemma 3.1).

When $\beta \notin \mathbb {N}$ we use the single index notation $C^{\beta }$ for the Hölder space $C^{\lfloor \beta \rfloor , \beta -\lfloor \beta \rfloor }$ where $\lfloor \cdot \rfloor$ denotes the integer part of a positive real number. Regarding Hölder norms of domains, we will assume throughout this paper that $B_2 \cap \Omega$ is a graph with $C^{\beta }$ norm bounded by one. We remark that this assumption does not incur in any loss of generality since any solution *u* to (1.2) can be rescaled as $u(rx)/r^\frac{2}{2-\gamma }$ to obtain a new solution in a domain with smaller Hölder norm where the graphicality assumption holds.

Finally, following a widely used notation, we will use *C* within proofs of propositions to indicate a unspecified constant, whose value will be allowed to change from line to line, and whose dependences will be clearly established in the statements of the corresponding propositions. We will make use of sub-indices whenever we will want to underline certain hidden dependencies of the constant.

## Regularized distance and first estimates

In our analysis we will use some classical properties of the distance function to the boundary and some of its generalizations. It is a classical result that, for $\beta \ge 1,$ nearby $C^\beta$ pieces of boundary, the distance function is $C^\beta .$ A priori the domains we are dealing with are $C^{1,\alpha },$ this forces us to consider regularized versions of the distance function.

### Lemma 2.1

Let $\beta >1$ with $\beta \notin \mathbb {N},$ and let $\partial \Omega \cap B_2$ be a $C^{\beta }$ graph. Then,  there exist a generalized distance function $d \in C^{\infty }( \Omega \cap B_1)\cap C^{\beta }({\overline{\Omega }}\cap B_1)$ satisfying

1. 2.1 $$\begin{aligned} \frac{1}{C} \text {dist}(x)\le d(x) \le C \text {dist}(x), \quad x\in \Omega \cap B_1, \end{aligned}$$
  where $$ \text {dist}(x):= \text {dist}(x, \partial \Omega ).$$
2. 2.2 $$\begin{aligned} d(x)= x_n +Q(x)+g(x), \quad x\in \Omega \cap B_1, \end{aligned}$$
  where $Q \in {\textbf{P}}_{\lfloor \beta \rfloor }$ with its zeroth and first order terms identically equal to zero and also satisfying $\Vert Q \Vert _{L^\infty (B_1)}\le C,$ and where $g \in C^{\infty }(\Omega \cap B_1)$ satisfies $|\nabla g| \le C|x|^{\beta -1}.$
3. For any $\lambda \in \mathbb {R},$ if $\beta \in (1,2)$
  2.3 $$\begin{aligned} |\Delta d^\lambda - \lambda (\lambda -1) d^{\lambda -2}|\le C d^{\beta +\lambda -3} \quad \text{ in } \Omega \cap B_1, \end{aligned}$$
  and if $\beta >2,$ then
  2.4 $$\begin{aligned} \Delta d^\lambda - \lambda (\lambda -1) d^{\lambda -2}= d^{\lambda -1}g(x), \quad \text{ in } \Omega \cap B_1, \end{aligned}$$
  with $$g \in C^{\beta -2}( {\overline{\Omega }}\cap B_1)\cap C^{\infty }( \Omega \cap B_1).$$
4. In particular,  we have that
  2.5 $$\begin{aligned} \Delta \Big ( c_\gamma d^\frac{2}{2-\gamma } \Big ) - \Big ( c_\gamma d^\frac{2}{2-\gamma } \Big )^{\gamma -1} = h \,\quad \text{ in } \Omega \cap B_1, \end{aligned}$$
  with $|h|\le Cd^{\beta +\frac{2}{2-\gamma }-3}$ if $\beta \in (1,2)$ and $h= d^\frac{\gamma }{2-\gamma }g(x)$ with $g \in C^{\beta -2}( {\overline{\Omega }}\cap B_1)$ if $\beta >2.$
5. If $$\beta \in (1,2)$$
  2.6 $$\begin{aligned} -\Delta \Big (d^\frac{1}{2} |\ln (d)|^\frac{1}{2}\Big )+ \frac{1}{4} \frac{d^\frac{2}{3} |\ln (d)|^\frac{1}{2}}{d^2} = \frac{1 }{4d^{\frac{3}{2}}|\ln (d)|^\frac{3}{2}}\Big (1 +h\Big ), \end{aligned}$$
  in $$ \cap \{d<1\}\cap B_1$$ with $$|h|\le C(\ln (d))^2d^{\beta -1}.$$

### Proof

Let us start addressing (1) and (2). We follow the construction in [29] (see also [18, Lemma B.0.1] considering

2.7 $$\begin{aligned} G(x,\rho ) = \int _{B_1} \text {dist}\Big (x-\frac{\rho }{2}z\Big )\phi (z)dz \end{aligned}$$

with $\phi \in C_c^\infty (B_1)$ a radial smooth non-negative function satisfying $\int _{B_1} \phi =1.$ Then *d* is defined implicitly by $d(x) = G(x,d(x)).$ In virtue of the implicit function theorem it follows that $d \in C^{\beta }( {\overline{\Omega }}\cap B_1)\cap C^{\infty }(\Omega \cap B_1).$ Additionally, (1) is a consequence of the construction as discussed in [29]. On the other hand, by differentiating the defining expression for *d* at a point $x\in \partial \Omega \cap B_2,$ we get

2.8 $$\begin{aligned} \nabla d(x) = \nabla \text {dist}(x) \int _{B_1} \phi (z)dz - \frac{1}{2}\nabla \text {dist}(x) \cdot \int _{B_1} z\phi (z)dz= \nabla \text {dist}(x), \end{aligned}$$

thanks to the properties of $\phi .$ Additionally, thanks to the uniform estimates on *d* and the orientation assumption on $\Omega$ (2) follows. We also remark that this construction satisfies

2.9 $$\begin{aligned} |\partial ^{\mu } d|\le C_{k} d^{\beta -|\mu |}, \text{ for } |\mu |>\beta . \end{aligned}$$

For part (3), a direct computation yields

2.10 $$\begin{aligned} \Delta d^\lambda = \lambda (\lambda -1)|\nabla d|^2 \frac{d^\lambda }{d^2}+\lambda d^{\lambda -1}\Delta d. \end{aligned}$$

So, if $\beta \in (1,2),$ we deduce from (2.8) that $|\nabla d(x)| =1$ on $\partial \Omega \cap B_1,$ which combined with the $C^\beta$ regularity implies that

2.11 $$\begin{aligned} \Big ||\nabla d|^2 - 1\big | \le C d^{\beta -1}\, \, \text{ in } \Omega \cap B_1. \end{aligned}$$

So, by combining (2.9), (2.10), and (2.11) we deduce (2.3). When $\beta >2,$ we have that the usual distance function satisfies (2.2) (see, e.g., [23, Section 14.6]), which is inherited by *d*. Hence, these considerations altogether with (1.5) imply (4), (4) realarly, we argue by a direct computation to show part (5). Given $\eta >0,$ from (2.3) and (2.9) we deduce

$$\begin{aligned} \Delta d^\lambda |\ln (d)|^\eta= &   \Delta (d^\lambda ) |\ln (d)|^\eta +2 \nabla (d^\lambda )\cdot \nabla |\ln (d)|^\eta +d^\lambda \Delta ( |\ln (d)|^\eta )\\= &   \Big (\lambda (\lambda -1) \frac{d^\lambda |\ln (d)|^\eta }{d^2}+ \Big (\frac{(\eta -1)}{|\ln (d)|}-2\lambda +1\Big )\frac{d^\lambda \eta |\ln (d)|^{\eta -1}}{d^2}\Big )|\nabla d|^2\\  &   + |\ln (d)|^\eta O( d^{\beta +\lambda -3})+|\ln (d)|^{\eta -1} O( d^{\beta +\lambda -3}) \end{aligned}$$

In particular, if $\lambda = \frac{1}{2}$ and $\eta =1/2,$ the previous expression together with (2.11) yields the expansion

$$\begin{aligned} \Delta \Big ( d^\frac{1}{2} |\ln (d)|^\frac{1}{2}\Big ) =\frac{1}{4} \frac{d^\frac{2}{3} |\ln (d)|^\frac{1}{2}}{d^2}-\frac{1 }{4d^{\frac{3}{2}}|\ln (d)|^\frac{3}{2}}\Big (1 +O(\ln (d)^2d^{\beta -1})\Big ), \end{aligned}$$

which concludes the proof. $\square$

In a similar vein, we construct the following barrier function that is still a sort of generalized distance satisfying other suitable properties.

### Lemma 2.2

Let $\rho \in (0,\frac{1}{2}),$ $\beta \in (1,2)$ and let $\partial \Omega \cap B_1$ be a $C^{\beta }$ graph. Then,  there exist a constant $C>0$ only depending on $\beta$ and $\rho ,$ and a function $\phi \in C^{\infty }(\Omega \cap B_1)\cap C^{\beta }( {\overline{\Omega }}\cap B_r)$ for any $r\in (0,1)$ such that

1. 2.12 $$\begin{aligned} \frac{1}{C} \text {d}(x)\le \phi (x) \le C \text {d}(x), \quad x\in \Omega \cap B_\rho , \end{aligned}$$
2. 2.13 $$\begin{aligned} \phi (x)\ge \frac{1}{C}\quad \text{ for } x \in \Omega \cap B_{2\rho }^c, \end{aligned}$$
3. 2.14 $$\begin{aligned} \phi (x)= x_n +g(x), \quad x\in \Omega \cap B_1, \end{aligned}$$
  where $g \in C^{\infty }(\Omega \cap B_2)$ satisfies $|\nabla g| \le C|x|^{\beta -1}$ in $\Omega \cap B_1,$
4. and for $\lambda >0$ we have that
  2.15 $$\begin{aligned} |\Delta \phi ^\lambda - \lambda (\lambda -1) \phi ^{\lambda -2}|\le C |x|^{\beta +\lambda -3}\quad \text{ in } \Omega \cap B_1. \end{aligned}$$

### Proof

Let $\phi _0$ be the unique solution of

2.16 $$\begin{aligned} {\left\{ \begin{array}{ll} \Delta \phi _0 = 0, &  \Omega \cap B_1,\\ \phi _0=\text {dist}(\cdot , B_{\rho }\cap \partial \Omega ), & \partial \big (\Omega \cap B_1 \big ), \end{array}\right. } \end{aligned}$$

where $\text {dist}$ is the standard distance function to a set. Let us notice that $\phi _0\in C^{\infty }( \Omega \cap B_1)\cap C^{\beta }({\overline{\Omega }}\cap B_r)$ for any $r \in (0,1).$ By the maximum principle $\phi _0>0$ in $\Omega \cap B_1.$ Thus, since $\phi _0$ vanishes tangentially along $\partial \Omega ,$ Hopf’s lemma together with the assumption $\nu _{\Omega }(0)= -e_n$ imply that $\nabla \phi (0) = C_{\Omega } e_n$ for some $C_{\Omega }$ satisfying $\frac{1}{C(\beta )}\le C_{\Omega } \le C(\beta )$ thanks to the uniform $C^{\beta }$ bound on $\Omega \cap B_1.$ Putting $\phi = \frac{1}{C_\Omega }\phi _0,$ we deduce (2.13) from standard regularity for harmonic functions [1, Lemma A.2], whereas (2.13) follows directly from the definition of $\phi _0.$

Part (3) follows from boundary Schauder estimates and the uniform bound on the $C^{\beta }$ norm of the domain. Lastly, for part (4) we argue as in Lemma 2.1

2.17 $$\begin{aligned} \Delta \phi ^\lambda = \lambda (\lambda -1)|\nabla \phi |^2 \frac{d^\lambda }{d^2}, \end{aligned}$$

where we have used the harmonicity of $\phi .$ So, by combining (2.17), with the fact that $|\nabla \phi (0)|=1$ and the expansion (2.14), we obtain (2.15). $\square$

In the following lemma, we recall some properties of solutions to (1.2) nearby regular points.

### Proposition 2.3

Let *u* be a solution to (1.2) and let $0 \in \partial \Omega$ be a regular free boundary point. Then,  there exists $r_0>0$ such that $\partial \Omega$ is $C^{1,\alpha }$ for any $\alpha \in (0,1).$ Moreover,  there exist $C, \rho >0$ (depending only on $\alpha , \gamma ,$ and *n*) such that

2.18 $$\begin{aligned} \Big |u(x)-c_\gamma d^\frac{2}{2-\gamma }(x)\Big | \le C |x|^{{\frac{2}{2-\gamma } + \alpha }} \quad \text{ for } x\in \Omega \cap B_{\rho }. \end{aligned}$$

Additionally, 

2.19 $$\begin{aligned} d(x)^{{\frac{\gamma }{2-\gamma }}}\le Cu_n(x) \quad \text{ for } x\in \Omega \cap B_{\rho }. \end{aligned}$$

### Proof

Let us start recalling that nearby 0,  $\partial \Omega$ is $C^{1,\alpha _0}$ for some $\alpha _0$ in virtue of the results in [4, 15]. Our goal is to show that $\alpha _0$ can be taken arbitrarily close to 1. We will show that this can be inferred from Appendix B in [19]—whose results, in that appendix, even if stated for $\gamma \in (0,1),$ hold true for the full range $\gamma \in (0,2)$ with exactly the same proofs.

Let us start deriving (2.18). Set $w:= \sqrt{\frac{\gamma }{2}}\beta v^\frac{2-\gamma }{2},$ which is normalized so that $|\nabla w(0)|=1,$ see [15]. Also, let us extend *w* as zero outside of $\Omega .$ Since 0 is a regular point for *u* and $\nu (0)=-e_n,$ given any $\varepsilon >0,$ we can find $\rho _\varepsilon$ such that

2.20 $$\begin{aligned} (x_n - C\rho _\varepsilon \varepsilon )_+ \le w(x) \le (x_n + C\rho _\varepsilon \varepsilon )_+, \quad { x\in B_{r}} \end{aligned}$$

where $C>0$ is a universal constant. Let $\alpha \in (0,1),$ in virtue of [19, Proposition B.2], if we select $\varepsilon _0>0$ (and in consequence $\rho _0)$ small enough in (2.20), we have that for any $r \in (0, \rho _0/2]$

$$\begin{aligned} |w(x) - (x\cdot e_0)_+ |\le C\varepsilon _0 r^{1+\alpha }, \quad { x\in B_{r}} \end{aligned}$$

where $C=C(\alpha , \gamma )$ and for some $e_0 \in {\mathbb {S}}^{n-1}.$ Equivalently, we have that

$$\begin{aligned} |w_r(x) - (x\cdot e_0)_+ |\le C\varepsilon r^{\alpha }, \quad { x\in B_{1}} \end{aligned}$$

where $w_r(x)= \frac{w(r x)}{r}.$ However, since 0 is a regular point for *u* and $\nu (0)=-e_n,$ we have that $w_r(x)\rightarrow (x_n)_+$ as $r\rightarrow 0^+,$ implying that $e_0=e_n$ and therefore

$$\begin{aligned} |w_r(x) - (x_n)_+ |\le C\varepsilon _0 r^{\alpha } \quad { x\in B_{1}} \end{aligned}$$

for $r \in (0, \rho _0/2).$ From this last inequality we easily deduce

2.21 $$\begin{aligned} |w(x) - (x_n )_+ |\le C|x|^{1+\alpha } \quad { x\in B_{\rho _0/2}.} \end{aligned}$$

Repeating the same argument in a neighborhood of 0,  we find $\rho _1>0$ such that for $z\in B_{\rho _1}\cap \partial \Omega$

2.22 $$\begin{aligned} \big |w(z+x) - (x \cdot (-\nu (z)) )_+ \big |\le C|x|^{1+\alpha } \quad { x\in B_{\rho _1}(z),} \end{aligned}$$

where $\nu (z)$ is the outer normal of $\partial \Omega$ at *z*. Thus, by applying (2.21) at the value $z\in B_{\rho _1/2}\cap \partial \Omega$ and (2.22) for this same *z* at the value $x-z$ for some $x \in B_{\rho _1/2},$ we deduce

2.23 $$\begin{aligned} \big | (x_n )_+-((x-z) \cdot (-\nu (z)) )_+ \big |\le &   |(x_n )_+-w(x)|+|w(x) - ( (x-z) \cdot (-\nu (z)) )_+ |\nonumber \\\le &   C\Big (|x|^{1+\alpha }+|z-x|^{1+\alpha }\Big ). \end{aligned}$$

On top of this, by plugging *z* in (2.21), we have that $|z \cdot e_n|= |(z_n)_+|\le C|z|^{1+\alpha }$ which combined with (2.23) yields

2.24 $$\begin{aligned} \big |(x-z)\cdot e_n -((x-z) \cdot (-\nu (z))_+ \big | \le C\Big (|x|^{1+\alpha }+|z-x|^{1+\alpha }+|z|^{1+\alpha }\Big ).\nonumber \\ \end{aligned}$$

On the other hand, in virtue of the flatness property (2.20) we have that

2.25 $$\begin{aligned} \{x \in B_{\rho _1} | x_n\ge C\varepsilon \rho _1 \} \subset \{w> 0\} \quad \text{ and } \quad |z_n|\le C\varepsilon |z|. \end{aligned}$$

Thus, upon taking $\varepsilon _0>0$ small enough in (2.20) (and thus shrinking further $\rho _1),$ given $y\in {\mathbb {S}}^{n-1}$ such that $y_n \ge \frac{1}{4}$ we can find $\{x \in B_{\rho _1} | x_n\ge C\varepsilon \rho _1 \}$ satisfying $|x|\le C_1 |z|\le C_2|x|$ such that $y=\frac{x-z}{|x-z|}.$ We observe now that in virtue of (2.25) and since the norms of |*x*| and |*z*| are comparable, we have that $|x|\le C_1 |z-x|\le C_2|x|.$ Additionally, thanks to the $C^{1,\alpha _0}$ of $\partial \Omega ,$ upon further shrinking $\rho _1,$ we that $(x-z)\cdot (-\nu (z))>0.$ Thus, by incorporating these considerations into (2.24), we deduce

$$\begin{aligned} \left| \frac{(x-z)}{|x-z|}\cdot (e_n -\nu (z)) \right| \le C|z|^{\alpha }, \end{aligned}$$

which in turn implies from our previous considerations

2.26 $$\begin{aligned} C\big | (e_n -\nu (z)) \big | = \sup _{ y \in {\mathbb {S}}^{n-1}, y_n \ge \frac{1}{4}} \big |y\cdot (e_n -\nu (z)) \big | \le C|z|^{\alpha }. \end{aligned}$$

From here we deduce that $\partial \Omega$ is $C^{1,\alpha }$ in $B_{\rho _1}.$

Aiming for (2.18), we can exploit the $C^{1,\alpha }$ regularity of $\partial \Omega \cap B_{\rho _1}$ to deduce from Lemma 2.1 that $d \in C^{1,\alpha }(\Omega \cap B_{\rho _1}).$ On top of this, since $\nabla d(0) =e_n,$ we have that $|d(x)-(x_n)_+|\le C|x|^{1+\alpha }$ for $x\in B_{\rho _1}.$ This combined with (2.21) implies

$$\begin{aligned} |w(x) - d(x)|\le C|x|^{1+\alpha } \quad { x\in \Omega \cap B_{\rho _1}.} \end{aligned}$$

From here, we can apply the mean value theorem to the function $t\rightarrow t^\frac{2}{2-\gamma }$ and recall the definition of $c_\gamma$ in (1.5) to deduce for $x\in B_{\rho _1}$

$$\begin{aligned} \left| u(x) - c_\gamma d^\frac{2}{2-\gamma }(x)\right|= &   C|w(x) - d(x)|\int _0^1 \big (tw(x)+(1-t)d(x)\big )^\frac{\gamma }{2-\gamma }\le C|x|^{\frac{2}{2-\gamma }+\alpha } \end{aligned}$$

where we have used that $u(x)\le Cd(x)^\frac{2}{2-\gamma }$ which, in turn, is consequence of [4, Corollary 1.11] combined with the boundary regularity of $\partial \Omega$ nearby 0.

Finally, since $w_n(0) =1,$ we deduce by continuity $\nabla w$ that $w_n \ge \frac{1}{C} u^\frac{1-\beta }{\beta } \ge \frac{1}{C} d^\frac{\gamma }{2-\gamma }$ on $\Omega \cap B_{\rho }$ for $\rho$ small enough. This shows (2.19) and finishes the proof. $\square$

## Boundary regularity for solutions of the linearized equation $L_\kappa$

As indicated in the introduction, our analysis relies on understanding the one dimensional solutions of (1.12). That is the purpose of the following lemma.

### Lemma 3.1

Let $\kappa \ge -\frac{1}{4}$ and $\theta _\pm$ defined as in (1.14). Given $c_i \in \mathbb {R}$ and $n_i \in \mathbb {N}\cup \{0\}$ for $i=1,2,$ with $c_2 =0$ if $\theta _-\le 0.$ Let *y* be a solution of

3.1 $$\begin{aligned} y''+ \frac{\kappa }{x^2}y=c_1 x^{\theta _++n_1-1}+c_2 x^{\theta _-+n_2-1}\quad \text{ in } (0,\infty ), \end{aligned}$$

satisfying

3.2 $$\begin{aligned} \begin{array}{rl} \displaystyle \lim _{x\rightarrow 0^+} \frac{y}{x^{\theta _-}} = 0 &  \quad \text {if}\ \kappa >-{\textstyle \frac{1}{4}} \\ \displaystyle \lim _{x\rightarrow 0^+} \frac{y}{x^{1/2} |\log x|} = 0 &  \quad \text {if}\ \kappa =-{\textstyle \frac{1}{4}} . \end{array} \end{aligned}$$

Then

3.3 $$\begin{aligned} y(x)= \lambda _1x^{\theta _+}+Ac_1 x^{\theta _++n_1+1}+B c_2 x^{\theta _-+n_2+1} \end{aligned}$$

where $\lambda _1 \in \mathbb {R}$ is any number,  and *A* and *B* are real valued parameters fully determined by $\kappa , n_1$ and $n_2.$

### Proof

The homogeneous ODE associated with (3.1) is a standard Cauchy–Euler equation whose solutions are of the form $x^\theta ,$ with $\theta$ satisfying

$$\begin{aligned} \theta (\theta -1)+\kappa =0. \end{aligned}$$

Therefore, characteristic roots correspond to

$$\begin{aligned} \theta _\pm = \frac{1\pm \sqrt{1+4 \kappa }}{2}, \end{aligned}$$

if $\kappa >\frac{-1}{4}.$ However, $x^{\theta _-}$ is discarded by the boundary condition (3.2). On the other hand, if $\kappa = -\frac{1}{4},$ we have multiplicity and thus we must replace $x^{\theta _-}$ by $\ln (x)x^\frac{1}{2}.$ This latter solution is also ruled out by (3.2). Finally, the structure of the particular solutions follows from standard computations in linear ODE theory, so we skip them for the sake of brevity. $\square$

### Boundary estimates

Our goal now is to prove Lemma 1.3 and derive uniform boundary estimates for solutions from it. We start showing an elementary form of the maximum principle for sub/super solutions of $L_\kappa$ with suitable boundary behavior.

#### Lemma 3.2

Let $D \subset \mathbb {R}^n$ be any bounded domain,  and let $v, \psi \in C^2(D)$ satisfying the following properties

3.4 $$\begin{aligned} L_\kappa v\le &   0, \quad \text{ in } D, \end{aligned}$$

3.5 $$\begin{aligned} L_\kappa \psi> &   0, \quad \text{ in } D,\nonumber \\ \psi> &   0, \quad \text{ in } D, \end{aligned}$$

and such that

3.6 $$\begin{aligned} \limsup _{x \rightarrow \partial D} \frac{v(x)}{\psi (x)} \le 0. \end{aligned}$$

Then,  $$v \le 0$$ in *D*.

#### Proof

Let us notice that by (3.6) either $v \le 0$ in *D* or $\frac{v}{\psi }$ attains a positive maximum inside *D*. Arguing by contradiction, we assume that the latter holds. This means that there is $M>0$ and $z\in D$ such that

$$\begin{aligned} v(z)= &   M \psi (z),\\ v\le &   M\psi , \quad \text{ in } \text{ D }. \end{aligned}$$

However, this latter observation yields $-\Delta v(z) \ge - M \Delta \psi (z),$ so we deduce that

$$\begin{aligned} 0\ge L_\kappa v(z) \ge M L_\kappa \psi (z) > 0, \end{aligned}$$

a contradiction. $$\square $$

As a corollary, we derive Lemma 1.3.

#### Proof Lemma 1.3

Let $\beta = 1+\alpha$ so that $\Omega \cap B_1$ is a $C^{\beta }$ domain with $\beta \in (1,2).$ Let $\rho >0$ such that $d<1$ on $\Omega \cap B_{\rho }$ and consider the function $\phi _1 = d^\frac{1}{2} (-\ln (d))^\frac{1}{2},$ which, thanks to (2.6), satisfies

3.7 $$\begin{aligned} L_\kappa \phi _1= &   \Big (\frac{1}{4}+\kappa \Big )\frac{ \phi _1}{d^2}+\frac{\phi _1 }{4d_1^{2}(-\ln (d_1))^2}\Big (1 +O(\ln (d)^2d^{\beta -1})\Big ). \end{aligned}$$

Since $d \le C\rho$ in $B_{\rho }\cap \Omega ,$ we can choose $\rho$ such that $1 +O(\ln (\rho )^2\rho ^{\min \{\beta -1,1\}}) \ge \frac{1}{2}$ in $\Omega \cap B_{\rho },$ depending only on the $C^{\beta }$ norm of $\Omega .$ Hence, from (3.7) we deduce

3.8 $$\begin{aligned} L_\kappa \phi _1 \ge \Big (\frac{1}{4}+\kappa \Big )\frac{ \phi _1}{d^2} +\frac{\phi _1}{d^2(-\ln (d_1))^2}(C-\rho ^{\beta -1}(-\ln (\rho ))^2)>0 \quad \text{ in } \Omega \cap B_{\rho },\nonumber \\ \end{aligned}$$

by shrinking $\rho$ further (if necessary) to make the last term positive. On the other hand, if $\kappa >-\frac{1}{4}$ (in which case we have that $\theta _- < \frac{1}{2}),$ we can combine (2.3) with the identity $\theta _-(\theta _- -1) =\kappa$ to derive the bounds

3.9 $$\begin{aligned} -C d^{\theta _--2}\rho ^{\beta -1}\le L_\kappa (d^{\theta _-})\le C d^{\theta _--2}\rho ^{\beta -1} \quad \text{ in } \Omega \cap B_{\rho }. \end{aligned}$$

Regardless of the value of $\kappa ,$ we can combine (3.8) and (3.9) to deduce that the barrier $\phi _2 = \phi _1+(1/4+\kappa )d^{\theta _-}$ satisfies

$$\begin{aligned} L_\kappa (\phi _2)> \big (d^{1/2-\theta _-} (-\ln (d))^\frac{1}{2}+O(\rho ^{\beta -1}) \big )\frac{ (1/4+\kappa ) d^{\theta _-}}{d^2}>0 \quad \text{ in } \Omega \cap B_{\rho }. \end{aligned}$$

Hence, if for some $r\le \rho ,$ we have that $v\le 0$ on $\Omega \cap \partial B_r ,$ we deduce that

$$\begin{aligned} \limsup _{x \rightarrow \partial (\Omega \cap B_r)} \frac{v(x)}{\phi _2(x)}\le 0, \end{aligned}$$

in virtue of the boundary condition (1.13). Finally, the result follows directly from Lemma 3.4. $\square$

We apply now the previous lemma to prove a first step towards Theorem 1.2, which will be improved later on. In virtue of this lemma, we will derive boundary estimates that will endow us with the required compactness to carry out our blow-up arguments.

#### Lemma 3.3

Let $\kappa \in [-\frac{1}{4},\infty ),$ let $\beta \in (1,2)$ and let $\Omega \subset \mathbb {R}^n$ be a $C^{\beta }$ domain. Let *d* be the regularized distance to $\partial \Omega ,$ given by Lemma 2.1.

Let $v \in C(\Omega \cap B_1)$ be any solution to

3.10 $$\begin{aligned} -\Delta v + \kappa \,\frac{v}{d^2}=f \quad \text {in}\ \Omega \cap B_1, \end{aligned}$$

satisfying

3.11 $$\begin{aligned} \begin{array}{rl} \displaystyle \lim _{x\rightarrow \partial \Omega } \frac{v}{d^{\theta _-}} = 0 &  \quad \text {if}\ \kappa >-{\textstyle \frac{1}{4}}\\ \displaystyle \lim _{x\rightarrow \partial \Omega } \frac{v}{d^{1/2} |\log d|} = 0 &  \quad \text {if}\ \kappa =-{\textstyle \frac{1}{4}}, \end{array} \end{aligned}$$

where $$f \in C(\Omega \cap B_1)$$ satisfies

3.12 $$\begin{aligned} f d^{2-\theta _+ -\varepsilon } \in L^\infty (\Omega \cap B_1 ) \end{aligned}$$

for some $$\varepsilon >0.$$

Then,  there exists $\rho \in (0,1)$ such that

3.13 $$\begin{aligned} |v| \le C\Big (\Vert v\Vert _{L^\infty (\Omega \cap B_1)}+ \Vert f \phi ^{2-\theta _+ -\varepsilon } \Vert _{L^\infty (\Omega \cap B_1)}\Big )d^{\theta _+},\quad \text{ in } \Omega \cap B_\rho , \end{aligned}$$

where $\phi$ is the barrier constructed in Lemma 2.2 and $\rho$ depends only on $\beta ,$ $\varepsilon$ and *n*.

Furthermore,  if $\kappa <0,$ then $v\in C^{\theta _+}({{\overline{\Omega }}}\cap B_{1/2})$ and

3.14 $$\begin{aligned} {[}v]_{\theta ^+, \Omega \cap B_\rho }\le C\Big (\Vert v\Vert _{L^\infty (\Omega \cap B_1)}+ \Vert f \phi ^{2-\theta _+ -\varepsilon }\Vert _{L^\infty (\Omega \cap B_1)}\Big ). \end{aligned}$$

Whilst,  if $\kappa \ge 0,$ we have the Lipschitz estimate

3.15 $$\begin{aligned} {[}v]_{1, \Omega \cap B_\rho }\le C\Big (\Vert v\Vert _{L^\infty (\Omega \cap B_1)}+ \Vert f \phi ^{2-\theta _+ -\varepsilon }\Vert _{L^\infty (\Omega \cap B_1)}\Big ). \end{aligned}$$

#### Proof

Let $\rho \in (0,1/2)$ to be determined later in the proof and let $\phi$ be the barrier constructed in Lemma 2.2. Thanks to (2.15), we can use the identity $(\theta _+-1)\theta _+ =\kappa$ as in (3.9) to deduce

3.16 $$\begin{aligned} |L_\kappa (\phi ^{\theta _+})|\le C \phi ^{\theta _+-2}\rho ^{\beta -1} \end{aligned}$$

in $$B_{2\rho } \cap \Omega .$$

Let $\varepsilon \in (0,\beta -1)$ such that (3.12) holds. Since $(\theta _++\varepsilon -1)(\theta _++\varepsilon )= \kappa +\varepsilon (2\theta _+-1+\varepsilon )$ and $\theta _+ \ge \frac{1}{2},$ we can proceed as in (3.16) to get

3.17 $$\begin{aligned} \Big | L_\kappa (\phi ^{\theta _++\varepsilon })+\varepsilon (2\theta _+-1+\varepsilon ) \phi ^{\theta _++\varepsilon -2}\Big | \le C\phi ^{\theta _++\varepsilon -2}\rho ^{\beta -1}, \end{aligned}$$

$$B_{2\rho } \cap \Omega .$$

So, we can fix $\rho >0$ small enough depending on $\varepsilon$ and $\beta$ such that for some $\eta >0$

3.18 $$\begin{aligned} L_k \Big (\phi ^{\theta _++\varepsilon }- \phi ^{\theta _+}\Big ) \le -\eta \phi ^{\theta ^++\varepsilon -2} \end{aligned}$$

in $B_{2\rho } \cap \Omega .$ Thus, after taking $\phi$ subordinated to $\rho ,$ we also have from (2.13) that

3.19 $$\begin{aligned} \phi ^{\theta _++\varepsilon }- \phi ^{\theta _+}\ge \eta \text{ on } \Omega \cap \partial B_{2\rho }. \end{aligned}$$

Let us divide both sides of (3.10) by

$$\begin{aligned} A=\frac{\Vert v \Vert _{L^\infty (\Omega \cap B_1)}+\Vert \phi ^{2-\theta _+ -\varepsilon } f \Vert _{L^\infty (\Omega \cap B_1)}}{\eta }, \end{aligned}$$

and let us notice that, by linearity, $v_1 =\frac{1}{A} v$ solves (3.10) with right hand side $f_1 =\frac{1}{A}f$ and with the same boundary condition (3.11). Furthermore, by construction $\Vert v_1 \Vert _{L^\infty (\Omega \cap B_1)}\le \eta$ and $|f_1|\le \eta \phi ^{\theta _+ +\varepsilon -2}$ in $\Omega \cap B_1.$

Thus, the function $u =v_1 -\Big (d^{\theta _+} +d^{\theta _+ +\varepsilon }\Big )$ satisfies the hypotheses of Lemma 1.3, implying that $v_1 \le \phi ^{\theta _+} -\phi ^{\theta _+ +\varepsilon }$ in $\Omega \cap B_{\rho }.$ By applying the previous argument to $-v$ and combining it with (2.12) we derive (3.13).

We complete the proof deducing (3.14). Let us consider first the case when $\kappa <0.$ Given $x_0 \in \Omega$ with $\text {dist}(x_0,\partial \Omega )= 2r<\rho ,$ we can combine (3.13) with interior Schauder estimates to deduce

$$\begin{aligned} {[} v ]_{C^{\theta _+}(B_\frac{r}{2}(x_0))}\le &   C\Big ( \frac{1}{r^{\theta _+}} \Vert v\Vert _{L^\infty (B_r(x_0))}+ r^{2-\theta _+} \left\| \frac{\kappa v}{d^2}-f\right\| _{L^\infty (B_r(x_0))}\Big )\\\le &   C\Big (\Vert v\Vert _{L^\infty (\Omega \cap B_1)}+ \Vert \phi ^{2-\theta _+ -\varepsilon } f \Vert _{L^\infty (\Omega \cap B_1)}\Big ). \end{aligned}$$

The estimate (3.14) follows from the previous inequality combined with a covering argument. See, e.g., Corollary 3.5 and Lemma B.2 in [28]. In the case $\kappa \ge 0,$ we can argue analogously to derive (3.15) see, e.g., [28, Corollary 3.5]. $\square$

#### Lemma 3.4

Let $M\in \mathbb {N},$ $\kappa \ge -\frac{1}{4},$ $\beta \in (1,2),$ and let $\Omega \subset \mathbb {R}^n$ be a $C^{\beta }$ domain. Let *d* be the regularized distance to $\partial \Omega ,$ given by Lemma 2.1. Let $v \in C(\Omega \cap B_1)$ be a solution of

3.20 $$\begin{aligned}  &   L_\kappa (v)(x) = \big (R_1(x)+g_1(x)\big )\left( \frac{d(r x)}{r}\right) ^{\theta _+-1} \nonumber \\  &   \quad +\big (R_2(x)+g_2(x)\big )\left( \frac{d(r x)}{r}\right) ^{\theta _--1}\quad \text {in}\ {\textstyle \frac{1}{r}}\Omega \cap B_1, \end{aligned}$$

with $R_i \in {\textbf{P}}_{M},$ and $g_i\in L^\infty ({\textstyle \frac{1}{r}}\Omega \cap B_1)$ for $i=1,2$ and with $R_2=g_2=0$ if $\theta _- \le 0.$ Then,  there exist $\rho _0>0$ and $C>0$ only depending on *M* and $\beta$ such that then for any $r \in (0,\rho _0),$

3.21 $$\begin{aligned} \Vert R_i\Vert _{L^\infty \big ({\textstyle \frac{1}{r}}\Omega \cap B_1\big )}\le C\big ( \Vert g_1\Vert _{L^\infty \big ({\textstyle \frac{1}{r}}\Omega \cap B_1\big )}+\Vert g_2\Vert _{L^\infty \big ({\textstyle \frac{1}{r}}\Omega \cap B_1\big )}+\Vert v\Vert _{L^\infty \big ({\textstyle \frac{1}{r}}\Omega \cap B_1\big )}\Big ),\nonumber \\ \end{aligned}$$

for $$i=1,2.$$

#### Proof

Arguing by contradiction, we assume sequences of domains $\Omega _l$ with $\Vert \partial \Omega _l \cap B_2\Vert _{C^\beta }\le 1,$ a sequence of radii $\{r_l\}_{l\in \mathbb {N}}$ with $r_l\rightarrow 0,$ and of polynomials $\{R_i^l\}_{l\in \mathbb {N}},$ functions $g_i^l \in L^\infty \big ( \frac{1}{r_l}\Omega \cap B_1\big )$ for $i=1,2$ and solutions $u_l$ to (3.20) such that (3.21) does not hold. Thus, by dividing both sides of (3.20) and (3.21) by $\Vert R_1^l\Vert _{L^\infty \big ( \frac{1}{r_l}\Omega \cap B_1\big )}+ \Vert R_2^l\Vert _{L^\infty \big ( \frac{1}{r_l}\Omega \cap B_1\big )}$ we obtain a sequence of functions $\{{\tilde{v}}_l\}$ satisfying

$$\begin{aligned} L_\kappa ({\tilde{v}}_l)(x)= &   \Big ({\tilde{R}}_1^l(x)+{\tilde{g}}_1^l(x)\Big )\Big (\frac{d_l(r x)}{r}\Big )^{\theta _+-1}\\  &   +\Big ({\tilde{R}}_2^l(x)+{\tilde{g}}_2^l(x)\Big )\Big (\frac{d_l(r x)}{r}\Big )^{\theta _--1},\quad \text {in}\ \frac{1}{r_l}\Omega \cap B_1, \end{aligned}$$

with $\Vert {\tilde{g}}_i^l\Vert _{L^\infty (\Omega \cap B_\rho )} \rightarrow 0$ as $l\rightarrow \infty$ for $i=1,2,$

3.22 $$\begin{aligned} \limsup _{l\rightarrow \infty } \Vert u_l\Vert _{L^\infty (B_1\cap \frac{1}{r_l}\Omega )}\rightarrow 0, \end{aligned}$$

and

$$\begin{aligned} \Vert {\tilde{R}}_1^l\Vert _{L^\infty \big ( \frac{1}{r_l}\Omega \cap B_1\big )}+ \Vert {\tilde{R}}_2^l\Vert _{L^\infty \big ( \frac{1}{r_l}\Omega \cap B_1\big )} =1. \end{aligned}$$

This last bound on the polynomials implies, bearing in mind that ${\textbf{P}}_{M}$ is a finite dimensional space, that, up to substracting a further subsequence, ${\tilde{R}}_i^l \rightarrow R^*_i$ for $i=1,2$ with at least one $Q_i^*\ne 0.$ On the other hand, in virtue of Lemma 2.1, we have that, up to subsequences,

$$\begin{aligned} \frac{d_l(r_l x)}{r_l}\rightarrow x_n, \end{aligned}$$

locally uniformly in $\mathbb {R}^n$ -by extending *d* by zero outside $\Omega \cap B_1.$ Therefore, by the stability of viscosity solutions under locally uniform convergence, we deduce that for $\lim _{l\rightarrow \infty } u_l=u_*=0$

$$\begin{aligned} 0= \Delta u_* - \frac{\tilde{C_\gamma }}{x_n^2}u = Q_1^* x_n^{\theta _+-1}+Q_2^* x_n^{\theta _--1}, \end{aligned}$$

which yields a contradiction since at least one of the $Q_i^*$ is not zero and since $\theta _+ -\theta _- \notin \mathbb {N}.$ $\square$

We prove now a Liouville-type theorem.

#### Lemma 3.5

Let $M \in \mathbb {N},$ $\kappa \ge -\frac{1}{4},$ $\theta _\pm$ as in (1.14),  and $\beta >1$ with $\beta \notin \mathbb {N}.$ Let $Q_i \in {\textbf{P}}_{M}$ for $i=1,2$ with $Q_2=0$ if $\theta _- \le 0.$ If *u* is such that

3.23 $$\begin{aligned} {\left\{ \begin{array}{ll} L_\kappa u = Q_1(x)x_n^{\theta _+-1}+Q_2(x)x_n^{\theta _--1} \quad \text {in} \ \{x_n>0\}\\ \Vert u\Vert _{L^\infty (B_R)} \le CR^{{\theta _+}+\beta -1}. \end{array}\right. } \end{aligned}$$

Let also us assume that *u* satisfies one of the following conditions

1. either
  3.24 $$\begin{aligned} \begin{array}{rl} \displaystyle \lim _{x_n \rightarrow 0^+} \frac{u(x',x_n)}{x_n^{\theta _-}} = 0,&  \quad \text {if}\ \kappa >-{\textstyle \frac{1}{4}}\\ \displaystyle \lim _{x_n \rightarrow 0^+} \frac{u(x',x_n)}{ \ln (x_n) x_n^\frac{1}{2}} = 0, &  \quad \text {if}\ \kappa =-{\textstyle \frac{1}{4}} . \end{array} \end{aligned}$$
2. or
  3.25 $$\begin{aligned} \lim _{x_n \rightarrow 0^+} \frac{u(x',x_n)}{x_n^{\theta _+}} = 0. \end{aligned}$$

Then,  $u = P_1(x)x_n^{\theta _+}+ P_2(x)x_n^{\theta _-+1},$ where $P_1 \in {\textbf{P}}_{\lfloor \beta \rfloor -1 }$ and $P_2 \in {\textbf{P}}_{\lfloor \beta +\theta _+-\theta - \rfloor -2 }.$ Furthermore,  if (3.24) holds, then $P_2=0$ whenever $Q_2=0.$ On the other hand,  if (3.25) holds,  $P_1=0$ whenever $Q_1=0.$

#### Proof

We will assume throughout the proof $\kappa <0,$ since the case $\kappa \ge 0$ is completely analogous.

Given any $R>1,$ let us define $u_R(x) = R^{-\theta _+-\beta +1}u(R x).$ Thanks to the growth bound in (3.23), we have that $\Vert u_R\Vert _{L^\infty (B_1)} \le C.$ Noticing that

$$\begin{aligned} L_k u_R = R^{2-\beta } Q_1(R x) x_n^{\theta _+-1}+ R^{2-\beta -\theta _++\theta _-} Q_2(R x) x_n^{\theta _--1}, \end{aligned}$$

we can use Lemma 3.4 in the flat domain $\Omega = \mathbb {R}^n_+,$ to deduce

3.26 $$\begin{aligned} R^{2-\beta }\Vert Q_1(R x)\Vert _{L^{\infty }(B_1)} + R^{2-\beta -\theta _++\theta _-}\Vert Q_2(R x)\Vert _{L^{\infty }(B_1)}\le C, \end{aligned}$$

for any $R>1,$ implying that

3.27 $$\begin{aligned} Q_1 \in {\textbf{P}}_{\lfloor \beta \rfloor -2}, \quad Q_2 \in {\textbf{P}}_{\lfloor \beta + \theta _+ -\theta _- \rfloor -2}. \end{aligned}$$

Now, taking $\varepsilon \in (0, 1-\theta _+-\theta _-)$ in (3.14), we deduce from (3.26) and Lemma 3.3 that $[ u_R ]_{C^{\theta _+}(B_\frac{1}{2})} \le C(1+\Vert Q_1\Vert _{L^\infty (B_1)}+\Vert Q_2\Vert _{L^\infty (B_1)}).$ This implies that

3.28 $$\begin{aligned} {[} u ]_{C^{\theta _+}\big (B_{R/2}\big )}\le C R^{\beta -1}. \end{aligned}$$

Arguing as in the proof of [1, Theorem 3.10], we consider the quotients

3.29 $$\begin{aligned} u_1(x) = \frac{u(x+\tau h)-u(x)}{h^{\theta _+}} \end{aligned}$$

with $h\in (0,R/2),$ $x\in \mathbb {R}^n_+,$ and $\tau \in {\mathbb {S}}^{n-1}$ with $\tau _n=0.$ From (3.28), we have that $\Vert u_1\Vert _{L^\infty (B_R)} \le CR^{\beta -1}.$ On the other hand, since $\tau$ is orthogonal to $e_n,$ we have that $u_1$ satisfies the boundary condition (3.24) together with

3.30 $$\begin{aligned} {\left\{ \begin{array}{ll} L_\kappa u_1 = {\tilde{Q}}_1(x)x_n^{\theta _+-1}+{\tilde{Q}}_2(x)x_n^{\theta _--1}, \quad \{x_n>0\}\\ \Vert u_1\Vert _{L^\infty (B_R)} \le CR^{\beta -1},\\ \end{array}\right. } \end{aligned}$$

where ${\tilde{Q}}_i = \frac{Q_i(x+\tau h)-Q_i(x)}{h^{\theta _+}}.$ Arguing as before, we conclude that $[ u_1 ]_{C^{\theta _+}\big (B_{R/2}\big )}\le C R^{\beta -1-\theta _+}.$ By repeating this argument inductively *k* times, until $k \theta _+ > \beta -1,$ we deduce that

$$\begin{aligned} {[} u_k ]_{C^{\theta _+}\big (B_{R/2}\big )}\le C R^{\beta -1- k \theta _+} \rightarrow 0, \end{aligned}$$

as $R\rightarrow \infty ,$ which, by the boundary condition, implies that $u_k =0.$ Therefore, we conclude iterating backwards the difference quotients (see the proof of [1, Theorem 3.10]) that

3.31 $$\begin{aligned} u(x',x_n) = \sum _{|\lambda |\le M} C_\lambda (x_n) (x')^\lambda , \end{aligned}$$

with $M = \lfloor \beta -1\rfloor$ for some functions $C_{\lambda }(x_n).$ Our next step consists in proving by induction that

$$\begin{aligned} C_\lambda (x_n)= P_{1,\lambda }(x_n)(x_n)_+^{\theta _+}+P_{2,\lambda }(x_n)(x_n)_+^{\theta _-+1}, \end{aligned}$$

for polynomials $P_{1,\lambda }$ and $P_{2,\lambda }$ satisfying, furthermore, that $P_{2,\lambda }(x_n)=0$ if $Q_2=0$ whenever (3.24) holds, and $P_{1,\lambda }(x_n)=0$ if $Q_1=0$ when we have (3.25) instead.

For the base case, if we take tangential derivatives of maximal order $\lambda _0,$ i.e., $|\lambda _0| = M,$ we conclude, bearing in mind (3.27), that $C_{\lambda _0}(x_n)$ satisfies

$$\begin{aligned} {\left\{ \begin{array}{ll} L_{\kappa ,1}(C_{\lambda _0})=0\\ C_{\lambda _0}(x_n)=0, \end{array}\right. } \end{aligned}$$

where $L_{\kappa ,1}(y) = -y''+\frac{\kappa }{x_n^2}y,$ i.e., the 1 dimensional version of $L_\kappa .$

Combining Lemma 3.1, and noticing from the decomposition (3.31) that $C_{\lambda }$ inherits the corresponding boundary condition from *u*,  we deduce that

$$\begin{aligned} C_{\lambda _0}(x_n)=C_{1,\lambda _0}x^{\theta _+}, \end{aligned}$$

with $C_{1,\lambda _0}=0$ if (3.24) holds instead. This proves the base case.

Let us assume now the result for $|\lambda |> j$ and show that it holds for $\beta$ with $|\beta |= j.$ Taking derivatives on both sides of (3.31) with respect to the tangential variables $x',$ and setting up the notation $v=\partial ^\beta u,$ we deduce from the inductive hypothesis that

3.32 $$\begin{aligned} u(x',x_n)= \beta ! C_{\beta }(x_n)+\sum _{\{ \lambda : \lambda >\beta \}} (x')^{\lambda -\beta }\Big ( P_{1,\lambda }(x_n)(x_n)_+^{\theta _+}+P_{2,\lambda }(x_n)_+^{\theta _-+1}\Big ),\nonumber \\ \end{aligned}$$

with $P_{i,\lambda }=0$ if $Q_i=0$ for $i=1,2,$ provided that the right corresponding boundary behavior is enforced. Thus, differentiating (3.23) and applying $L_{\kappa ,1}$ to the representation (3.32) yields

3.33 $$\begin{aligned} \beta ! L_{k,1}(C_{\beta }(x_n))= &   \partial ^\beta Q_1(x)x_n^{{\theta _-}-1}+ \partial ^\beta Q_2(x)x_n^{{\theta _+}-1}\nonumber \\  &   - \sum _{\{ \lambda : \lambda>\beta \}} (\Delta (x')^{\lambda -\beta })\Big ( P_{1,\lambda }(x_n)x_n^{{\theta _+}}+P_{2,\lambda }(x_n)x_n^{\theta _-+1}\Big )\nonumber \\  &   - \sum _{\{ \lambda : \lambda >\beta \}} ( (x')^{\lambda -\beta })\Big ( P_{1,\lambda }''(x_n)x_n^{\theta _+}+P_{2,\lambda }''(x_n)x_n^{\theta _-}\Big ). \end{aligned}$$

Therefore, since $L_{\kappa ,1}(C_{\beta }(x_n))$ does not depend on $x',$ we deduce from (3.33) that

3.34 $$\begin{aligned} L_{\kappa ,1}(C_{\beta }(x_n))={\tilde{P}}_{1}(x_n)x_n^{{\theta _+}-1}+ {\tilde{P}}_{2}(x_n)(x_n)x_n^{{\theta _-}-1}, \end{aligned}$$

for some polynomials ${{\tilde{P}}_{1}}$ and ${\tilde{P}}_{2},$ with ${\tilde{P}}_{i}=0$ if $Q_i =0$ for $i=1,2.$ Then, combining again Lemma 3.1, with either (3.24) or (3.25), the decomposition (3.31), and the linearity of $L_{\kappa ,1}$ we deduce

$$\begin{aligned} C_{\beta }(x_n) = P_{1,\beta }(x_n)x_n^{\theta _+}+ P_{2,\beta }(x_n)x_n^{\theta _-+1}, \end{aligned}$$

with $P_{i,\beta }=0$ if $Q_i =0$ for $i=1,2.$

This completes the inductive argument implying

3.35 $$\begin{aligned} u(x) = P_1(x)x_n^{\theta _+}+ P_2(x)x_n^{\theta _-+1} \end{aligned}$$

for some polynomials $$P_1$$ and $$P_2,$$ with $$ P_{i}=0$$ if $$Q_i =0.$$

Lastly, since $\theta _+-\theta _- \notin \mathbb {N},$ each term in (3.35) must satisfy the same growth bound as *u*. This implies that $P_1 \in {\textbf{P}}_{\lfloor \beta \rfloor -1}$ and that $P_2 \in {\textbf{P}}_{\lfloor \beta +\theta _+-\theta - \rfloor -2 },$ finishing the proof. $\square$

## Higher order boundary Schauder estimates for solutions to the linearized equation

This section is devoted to the proof of a generalized version of Theorem 1.2, which is carried out using a blow-up in the same spirit of [1]. We divide the argument into two parts, addressing first the case where $\partial \Omega \cap B_1$ is a $C^{\beta }$ surface with $\beta \in (1,2)$ and then proving the higher order version of this result.

### Theorem 4.1

Let $\kappa \ge -\frac{1}{4},$ $\theta _\pm$ as in (1.14),  and $\beta \in (1,2)$ with $\beta +\theta _+-\theta _- \notin \mathbb {N}.$ Let $\Omega \subset \mathbb {R}^n$ such that $\partial \Omega \cap B_2$ a $C^{\beta }$ surface,  let *f* and *g* satisfying $\Vert f\Vert _{C^{\beta -1}({\overline{\Omega }} \cap B_1)}, \Vert g\Vert _{C^{\beta -1+\theta _+-\theta _-}({\overline{\Omega }} \cap B_1)}\le 1$ and $f, g|_{\partial \Omega }\equiv 0,$ where $g=0$ if $\theta _- \le 0.$ Let $v \in C(\Omega \cap B_1)$ be a solution to

4.1 $$\begin{aligned} L_\kappa (v)= fd^{\theta _+-2}+gd^{\theta _--2},\quad \text {in}\ \Omega \cap B_1, \end{aligned}$$

satisfying

4.2 $$\begin{aligned} \begin{array}{rl} \displaystyle \lim _{x\rightarrow \partial \Omega } \frac{v}{d^{\theta _-}} = 0 &  \quad \text {if}\ \kappa >-{\textstyle \frac{1}{4}} \\ \displaystyle \lim _{x\rightarrow \partial \Omega } \frac{v}{d^{1/2} |\log d|} = 0 &  \quad \text {if}\ \kappa =-{\textstyle \frac{1}{4}}. \end{array} \end{aligned}$$

Then,  there exists a constant $C>0$ and a radius $\rho >0,$ such that for every $x_0 \in \partial \Omega \cap B_\rho ,$ there are constants $\lambda _{1,x_0}, \lambda _{2,x_0} \in \mathbb {R}$ such that

4.3 $$\begin{aligned}  &   |v(x)-\lambda _{1,x_0}d^{\theta _+}(x)-\lambda _{2,x_0}(x)d^{\theta _-+1}(x)| \le C |x-x_0|^{{{\theta _+} + \beta -1}}\quad \text{ for } x\in B_{\rho }(x_0)\cap \Omega ,\nonumber \\ \end{aligned}$$

with $\lambda _{2,x_0}=0$ if $g=0.$ Here the constants *C* and $\rho$ depend only on $\kappa ,$ $\beta ,$ and *n*.

### Proof

*Preparations:* Without loss of generality, let us take $x_0=0$ and let us extend *v* and *d* by zero outside $\Omega \cap B_1.$ So, if we contradict the inequality (4.3), we deduce the existence of a sequence of domains $\Omega _j$ with $\Vert \Omega _j\Vert _{C^{\beta }}\le 1$ and, in consequence, of generalized distance functions $d_j$ such that for any sequence of constants $\lambda _{i,j} \in \mathbb {R}$ (with $k_{2,j}=0$ if $\theta _- \le 0),$ we have that

4.4 $$\begin{aligned} \sup _{r >0} \sup _{j\in \mathbb {N}} \frac{1}{r^{{{\theta _+} + \beta -1}}} \Vert v_j-\lambda _{1,j} d_j^{\theta _+}- \lambda _{2,j}d_j^{\theta _-+1}\Vert _{L^\infty (B_r)}=\infty , \end{aligned}$$

where $v_j$ satisfies (4.2) and

4.5 $$\begin{aligned} L_\kappa (v_j)= f_jd^{\theta _+-2}+g_jd^{\theta _--2},\quad \text {in}\ \Omega \cap B_1, \end{aligned}$$

with $\Vert f_{j}\Vert _{C^{\beta -1}(B_1\cap \overline{\Omega _j})}, \Vert g_{j}\Vert _{C^{\beta -1+\theta _+-\theta _-}(B_1\cap \overline{\Omega _j})}\le 1$ with $f_j, g_j|_{\partial \Omega _j}\equiv 0$ for $j\in \mathbb {N}$ and with $g_{j}=0$ if $\theta _- \le 0.$

*Step one:* We start selecting $\lambda _{1,j,r}, \lambda _{2,j,r}$ as the least square coefficients of the projection of $v_j$ onto the subspace spanned by $\{d_j^{\theta _+},d_j^{\theta _-+1}\}$ in $L^2(B_r).$ In this case, they are characterized by the orthogonality conditions

4.6 $$\begin{aligned} \int _{B_r} \Big (v_j -\lambda _{1,j,r}d_j^{\theta _+}- \lambda _{2,j,r}d_j^{\theta _-+1}\Big )(c_1d_j^{\theta _+}+c_2d_j^{\theta _-+1})=0, \end{aligned}$$

for any $$(c_1, c_2)\in \mathbb {R}^2.$$

Let us define

$$\begin{aligned} \theta (r) = \sup _{\rho >r }\sup _{j\in \mathbb {N}} \frac{1}{r^{{{\theta _+} + \beta -1}}} \big \Vert v_j -\mu _{1,j} d_j^{\theta _+}- \mu _{2,j} d_j^{\theta _-+1}\big \Vert _{L^\infty (B_r)}, \end{aligned}$$

with $$\mu _{1,j}= \lambda _{1,j,r}$$ and $$\mu _{2,j}= \lambda _{2,j,r}.$$

By Lemma 6.2 we have that $\lim _{r\rightarrow 0^+} \theta (r)=\infty .$ Thus, we can pick a sequence $\{r_j\}_{j\in \mathbb {N}}$ with $r_j\rightarrow 0^+$ as $j\rightarrow \infty$ such that

4.7 $$\begin{aligned} w_{j}(x):= \frac{ v_{j}(r_j x)-\mu _{1,j}d_j^{\theta _+}(r_j x)-\mu _{2,j}d_j^{\theta _-+1}(r_j x)}{r_j^{{{\theta _+} +\beta -1}}\theta (r_j)}, \end{aligned}$$

satisfies $$ \Vert w_j\Vert _{L^\infty (B_1)} \in [\frac{1}{2},1]$$ for $$l\in \mathbb {N}.$$

Additionally, Lemma 6.3 provides the growth bound

4.8 $$\begin{aligned} \Vert w_j \Vert _{L^\infty (B_R)}\le C R^{{\theta _+}+\beta -1}. \end{aligned}$$

*Step two:* We proceed to show that the sequence $\{w_j\}_{j\in \mathbb {N}}$ satisfies, up to a small perturbation, a PDE of the form (3.10). Applying Lemma 2.1, we can easily compute the Laplacian of the auxiliary term $\mu _{1,j}d_j^{\theta _+}(r_jx)+\mu _{2,j}d_j^{\theta _-+1}(r_jx)$ as follows

4.9 $$\begin{aligned}  &   \frac{1}{r_j^{{\theta _+}+\beta -1}}\Delta \Big (\mu _{1,j}d_j^{\theta _+}(r_jx)+\mu _{2,j}d_j^{\theta _-+1}(r_j x)\Big )\nonumber \\  &   \quad =\frac{1}{r_j^{\theta _++\beta -1}}\frac{r_j^2\kappa }{d_j^2(rx)}\Big (\mu _{1,j} d_j^{\theta _+}(r_jx)+\mu _{2,j}d_j^{\theta _-+1}(r_jx)\Big )\nonumber \\  &   \qquad + l_j \Big ( \frac{d_j(rx)}{r}\Big )^{\theta _--1}+ h_{1,j}(x) \Big ( \frac{d_j(rx)}{r}\Big )^{\theta _++\beta -3}\nonumber \\  &   \qquad + h_{2,j}(x) \Big ( \frac{d_j(rx)}{r}\Big )^{\theta _-+\beta -2} \end{aligned}$$

where $l_j \in \mathbb {R},$ $\Vert h_{1,j}\Vert _{L^\infty (B_2\cap \overline{\Omega _j})}\le \frac{C}{\theta (r_j)}$ and $\Vert h_{2,j}\Vert _{L^\infty (B_2\cap \overline{\Omega _j})}\le \frac{C r_j^{\theta _--\theta _++1}}{\theta (r_j)}.$ We remark that the latter quantity goes to zero as $j\rightarrow \infty$ since $\theta _+-\theta _- \le 1.$

By combining (4.9) with (4.5), we deduce

4.10 $$\begin{aligned} L_\kappa ( w_j)= &   l_j \Big ( \frac{d_j(rx)}{r}\Big )^{\theta _--1}+\nonumber \\  &   + h_{1,j}(x) \Big ( \frac{d_j(rx)}{r}\Big )^{\theta _++\beta -3}+ h_{2,j}(x) \Big ( \frac{d_j(rx)}{r}\Big )^{\theta _-+\beta -2} \end{aligned}$$

4.11 $$\begin{aligned}  &   + {\tilde{f}}_j(x) \Big (\frac{d_j(r_jx)}{r_j}\Big )^{\theta _+-2}+{\tilde{g}}_j(x)\Big (\frac{d_j(rx)}{r}\Big )^{\theta _--2} \end{aligned}$$

where ${\tilde{f}}_{j}= \frac{f_j(r_j x)}{\theta (r_j)r_j^{\beta -1}}$ and ${\tilde{g}}_{j}= \frac{g_j(r_j x)}{\theta (r_j)r_j^{\beta +\theta _+-\theta _--1}}$ and with both vanishing uniformly as $j\rightarrow \infty$ thanks to the hypotheses on $f_j$ and $g_j.$ In particular, we are fundamentally exploiting that $|f_j|\le C d_j^{\beta -1}$ and that $|g_j|\le Cd_j^{\beta -1+\theta _+-\theta _-}.$

On the other hand, let us notice that the hypotheses (4.2) force $w_j$ to satisfy the boundary conditions (3.11). With the aim of applying Lemma 3.3, we take $\varepsilon \in (0,\min \{\beta -1, 1+\theta _--\theta _+\})$ in (3.13) so that the right hand side in (4.104.11) remains uniformly bounded after multiplying by $d_j^{2-\theta _+-\varepsilon }.$ Thus, in virtue of Lemmas 3.3 and 3.4, the solutions $\{w_j\}_{j\in \mathbb {N}}$ to the sequence of problems (4.104.11) are equicontinuous and, therefore, $w_j \rightarrow w_0$ locally uniformly as $j\rightarrow \infty$ -up to subsequences. So, thanks to Lemma 3.4, and (4.8) we deduce that

4.12 $$\begin{aligned} {\left\{ \begin{array}{ll} L_\kappa w_0 = l x_n^{\theta _--1}, \quad \text{ in } \{x_n>0\},\\ \Vert w_0\Vert _{L^\infty (B_R\cap \mathbb {R}^n_+)} \le CR^{{\theta _+}+\beta -1}. \end{array}\right. } \end{aligned}$$

Additionally, thanks to the uniform convergence, the inequality (3.13) is satisfied by each $w_j$ implies that

4.13 $$\begin{aligned} |w_0|\le C x_n^{\theta _+}. \end{aligned}$$

*Step 3:* Since $w_0$ satisfies (4.12) and (4.13), Lemma 3.5 applies, implying that

$$\begin{aligned} w_0= \lambda _1 (x_n)_+^{\theta _+}+\lambda _2 (x_n)_+^{\theta _-+1} \end{aligned}$$

with $\lambda _1,\lambda _2 \in \mathbb {R}.$ However, taking limits in the orthogonality conditions (4.6) as $j\rightarrow \infty ,$ yields

$$\begin{aligned} \int _{B_1} w_0 \Big (c_1(x_n)_+^{\theta _+}+c_2(x_n)_+^{\theta _-+1}\Big )=0, \end{aligned}$$

for any $(c_1, c_2)\in \mathbb {R}^2.$ Thus, $w_0=0$ contradicting that $\Vert w_0\Vert _{L^\infty (B_1)}\ge \frac{1}{2}.$ $\square$

In the context of in Theorem 1.2, if $\partial \Omega \cap B_1 \in C^{\beta }$ with $\beta >2,$ since $f|_{\partial \Omega }\equiv 0,$ we have that $fd^{\mu -2}={\tilde{f}}d^{\mu -1}$ where ${{\tilde{f}}} = f/d$ is one degree less regular than *f* up to the boundary. Based on this observation, we prove the following higher order version of Theorem 4.1.

### Theorem 4.2

Let $\kappa \ge -\frac{1}{4},$ $\theta _\pm$ as in (1.14),  let $\beta >2$ with $\beta \notin \mathbb {N}.$ Let $\Omega \subset \mathbb {R}^n$ with $\partial \Omega \cap B_1$ a $C^{\beta }$ surface,  let *f* and *g* satisfying $\Vert f\Vert _{C^{\beta -2}({\overline{\Omega }} \cap B_1)}, \Vert g\Vert _{C^{\beta -2+\theta _+-\theta _-}({\overline{\Omega }} \cap B_1)}\le 1,$ with $g=0$ if $\theta _- \le 0.$ Let $v \in C({\overline{\Omega }}\cap B_1)$ be a solution to

4.14 $$\begin{aligned} L_\kappa (v)= fd^{\theta _+-1}+g d^{\theta _--1},\quad \text {in}\ \Omega \cap B_1, \end{aligned}$$

satisfying

4.15 $$\begin{aligned} \begin{array}{rl} \displaystyle \lim _{x\rightarrow \partial \Omega } \frac{v}{d^{\theta _-}} = 0 &  \quad \text {if}\ \kappa >-{\textstyle \frac{1}{4}} \\ \displaystyle \lim _{x\rightarrow \partial \Omega } \frac{v}{d^{1/2} |\log d|} = 0 &  \quad \text {if}\ \kappa =-{\textstyle \frac{1}{4}}. \end{array} \end{aligned}$$

Then,  there exists a constant $C>0$ and a radius $\rho >0,$ such that for every $x_0 \in \partial \Omega \cap B_\rho ,$ there exists polynomials $Q_{1,x_0} \in {\textbf{P}}_{\lfloor \beta \rfloor -1}$ and $Q_{2,x_0} \in {\textbf{P}}_{\lfloor \beta +\theta _+-\theta _- \rfloor -2 }$ such that

4.16 $$\begin{aligned}  &   \Big |v(x)-Q_{1,x_0}(x)d^{\theta _+}(x)-Q_{2,x_0}(x)d^{\theta _-+1}(x)\Big | \nonumber \\  &   \quad \le C |x-x_0|^{{{\theta _+} + \beta -1}}\quad \text{ for } x\in B_{\rho }(x_0)\cap \Omega , \end{aligned}$$

with $$Q_{2,x_0}=0$$ if $$g=0.$$

### Proof

*Preparations:* Let us assume again $x_0=0$ and let us extend *v* and *d* by zero in $\Omega ^c\cap B_1.$ By contradicting the inequality (4.16), we deduce the existence of a sequence of domains $\Omega _j$ with $\Vert \Omega _j\Vert _{C^{\beta }}\le 1$ and, in consequence, of generalized distance functions $d_j$ such that for any sequence of polynomials $P_{1,j} \in {\textbf{P}}_{\lfloor \beta \rfloor -1}$ and $P_{2,j} \in {\textbf{P}}_{\lfloor \beta +\theta _+-\theta _- \rfloor -1}$

4.17 $$\begin{aligned} \sup _{r >0} \sup _{j\in \mathbb {N}} \frac{1}{r^{{{\theta _+} + \beta -1}}} \Big \Vert v_j-P_{1,j} d_j^{\theta _+}- P_{2,j}d_j^{\theta _-+1}\Big \Vert _{L^\infty (B_r)}=\infty , \end{aligned}$$

with $v_j$ satisfying (4.15) and

4.18 $$\begin{aligned} L_\kappa (v_j)= f_{1,j}d_j^{\theta _+-1}+f_{2,j}d_j^{\theta _--1}\quad \text {in}\ \Omega \cap B_1 \end{aligned}$$

and where $\Vert f_{1,j}\Vert _{C^{\beta -2}(B_2\cap \overline{\Omega _j})}\le 1$ and $\Vert f_{2,j}\Vert _{C^{\beta +\theta _+-\theta _- -2}(B_2\cap \overline{\Omega _j})}\le 1$ for $j \in \mathbb {N}.$ Furthermore, we take $f_{2,j}= P_{2,j}=0$ if $g=0.$

*Step one:* We select the least squares polynomials ${(Q_{1,j,r}, Q_{2,j,r}) \in {\textbf{P}}_{\lfloor \beta \rfloor -1}}$ ${\times {\textbf{P}}_{\lfloor \beta +\theta _+-\theta _- \rfloor -2}},$ which are characterized by the orthogonality conditions

4.19 $$\begin{aligned} \int _{B_r} (v_j -Q_{1,j,r} d_j^{\theta _+}- Q_{2,j,r}d_j^{\theta _-+1})(P_1(x)d_j^{\theta _+}+P_2(x)d_j^{\theta _-+1})=0, \end{aligned}$$

for any $(P_1, P_2)\in {\textbf{P}}_{\lfloor \beta \rfloor -1} \times {\textbf{P}}_{\lfloor \beta +\theta _+-\theta _- \rfloor -2}$ for $r>0$ and $j\in \mathbb {N}.$

Let us define

$$\begin{aligned} \theta (r) = \sup _{\rho >r }\sup _{j\in \mathbb {N}} \frac{1}{r^{{{\theta _+} + \beta -1}}} \Big \Vert v_j -Q_{1,j,r} d_j^{\theta _+}- Q_{2,j,r}d_j^{\theta _-+1}\Big \Vert _{L^\infty (B_r)}. \end{aligned}$$

By Lemma 6.2 we have that $\lim _{r\rightarrow 0^+} \theta (r)=\infty .$ Thus, we can pick a sequence $\{r_j\}_{j\in \mathbb {N}}$ with $r_j\rightarrow 0^+$ as $j\rightarrow \infty$ such that

4.20 $$\begin{aligned} w_{j}(x):= \frac{ v_{j}(r_j x)-Q_{1,j,r_j}(r_jx)d_j^{\theta _+}(r_j x)-Q_{2,j,r_j}(r_jx)d_j^{\theta _-+1}(r_j x)}{r_j^{{{\theta _+} +\beta }}\theta (r_j)},\nonumber \\ \end{aligned}$$

satisfies $$ \Vert w_j\Vert _{L^\infty (B_1)} \in [\frac{1}{2},1]$$ for $$j\in \mathbb {N}.$$

Additionally, Lemma 6.3 provides the growth bound

4.21 $$\begin{aligned} \Vert w_j \Vert _{L^\infty (B_R)}\le C R^{{\theta _+}+\beta -1}. \end{aligned}$$

*Step two:* We proceed to show that the sequence $\{w_j\}_{j\in \mathbb {N}}$ satisfies, up to a small perturbation, a PDE of the form (3.10). Let us start noticing that since we fall under the hypothesis of Lemma 6.3, we can estimate the Laplacian of the auxiliary term $Q_{1,j,r} d_j^{\theta _+}+ Q_{2,j,r}d_j^{\theta _-+1}$ as follows

4.22 $$\begin{aligned}  &   \frac{1}{\theta (r)r^{{\theta _+}+\beta -1}}\Delta \Big (Q_{1,r}(rx)d_j^{\theta _+}(r x)+Q_{2,r}(rx)d_j^{\theta _-+1}(r x)\Big ) \nonumber \\  &   \quad =\frac{1}{\theta (r)r^{{\theta _+}+\beta -1}}\frac{\kappa r^2}{d_j^2(rx)}Q_{1,r}(rx)d_j^{\theta _+}(r x) \nonumber \\  &   \qquad +\frac{1}{\theta (r)r^{{\theta _+}+\beta -1}}\frac{\kappa r^2}{d_j^2(rx)}Q_{2,r}(rx)d_j^{\theta _-+1}(r x)\nonumber \\  &   \qquad +P_{1,r}(x)\Big ( \frac{d_j(rx)}{r}\Big )^{\theta _+-1}\nonumber \\  &   \qquad +P_{2,r}(rx) \Big ( \frac{d_j(rx)}{r}\Big )^{\theta _--1}\nonumber \\  &   \qquad +m_*(r,x) \end{aligned}$$

where $P_{i,r} \in {\textbf{P}}_{M}$ for $i=1,2$ for some $M \in \mathbb {N}$ only depending on $\beta$ and $\kappa$ and where $|m_*(x,r)|\le Cm(r)$ with *m* depending only on $\beta$ and $\kappa$ and such that $m(r)\rightarrow 0$ as $r\rightarrow 0^+.$

By combining (4.18), (4.22), and exploiting the uniform regularity of the functions $f_{i,j}$ we deduce

4.23 $$\begin{aligned} L_\kappa ( w_j)= &   \Big ( {\tilde{Q}}_{1,r}(x)+g_{1,j}(x) \Big )\Big ( \frac{d_j(rx)}{r}\Big )^{\theta _+-1} \nonumber \\  &   +\Big ( {\tilde{Q}}_{2,r}(rx) +g_{2,j}(x)\Big ) \Big ( \frac{d_j(rx)}{r}\Big )^{\theta _--1}\nonumber \\  &   +g_{3,j}(x), \end{aligned}$$

where ${\tilde{Q}}_{i,j} \in {\textbf{P}}_{M }$ for some $M\in \mathbb {N}$ and $\Vert g_{i,j} \Vert _{L^\infty (B_2\cap \overline{\Omega _j})}\rightarrow 0$ as $j\rightarrow \infty$ for $i=1,2,3.$

On the other hand, let us notice that the hypotheses (4.15) force $w_j$ to satisfy the boundary conditions (3.11). With the aim of applying Lemma 3.3, we take $\varepsilon \in (0,1+\theta _--\theta _+)$ in (3.13) so that the right hand side in (4.23) remains uniformly bounded after multiplying by $d_j^{2-\theta _+-\varepsilon }.$ Thus, in virtue of Lemma 3.4 and Lemma 3.3, the solutions $\{w_j\}_{j\in \mathbb {N}}$ to the sequence of problems (4.104.11) are equicontinuous and, therefore, satisfy that $w_j \rightarrow w_0$ locally uniformly. So, thanks to Lemma 3.4, and (4.8) we deduce that

4.24 $$\begin{aligned} {\left\{ \begin{array}{ll} L_\kappa w_0 = Q_1(x)(x_n)_+^{\theta _+-1}+Q_2(x)(x_n)_+^{\theta _--1} \quad \text{ in } \{x_n>0\},\\ \Vert w_0\Vert _{L^\infty (B_R\cap \mathbb {R}^n_+)} \le CR^{{\theta _+}+\beta -1} \end{array}\right. } \end{aligned}$$

with $Q_i \in {\textbf{P}}_{M}$ for some $M\in \mathbb {N}$ and for $i=1,2$ and with $Q_2=0$ if $g=0.$ Additionally, thanks to the uniform convergence, the inequality (3.13) satisfied by each $w_j$ implies that

4.25 $$\begin{aligned} |w_0|\le C x_n^{\theta _+}. \end{aligned}$$

*Step 3:* Since $w_0$ satisfies (4.24) and (4.25), Lemma 3.5 applies, implying that

$$\begin{aligned} w_0= P_1(x)(x_n)_+^{{\theta _+}}+P_2(x)(x_n)_+^{{\theta _-+1}}, \end{aligned}$$

with $P_1 \in {\textbf{P}}_{\lfloor \beta \rfloor -1}$ and $P_2 \in {\textbf{P}}_{\lfloor \beta +\theta _+-\theta _-\rfloor -2},$ with $P_2=0$ if $g=0.$ However, taking limits in the orthogonality condition (4.19) as $j\rightarrow \infty ,$ yields

$$\begin{aligned} \int _{B_1} w_0 \Big (Q_1(x)x_n^{{\theta _+}}+Q_2(x)x_n^{{\theta _-+1}}\Big )=0, \end{aligned}$$

for all $Q_1 \in {\textbf{P}}_{\lfloor \beta \rfloor -1}$ and $Q_2 \in {\textbf{P}}_{\lfloor \beta +\theta _+-\theta _-\rfloor -2}.$ Thus, $w_0=0$ contradicting $\Vert w_0\Vert _{L^\infty (B_1)}\ge \frac{1}{2}.$ $\square$

The following technical lemma shows how to translate estimates of the form (4.16) into regularity for the quotients $v/d^\mu .$

### Lemma 4.3

Let $\mu >0,$ let $\beta >1$ with $\beta \notin \mathbb {N},$ and let $\Omega \subset \mathbb {R}^n$ with $\partial \Omega \cap B_1$ a $C^{\beta }$ surface. Let $v \in C^{\beta }(\Omega \cap B_1)$ and let us assume that for every $z \in \Omega \cap B_1$ there exists $Q_z \in {\textbf{P}}_{\lfloor \beta -1\rfloor }$ such that for every $y_0 \in B_\frac{1}{2}(z)\cap \Omega$ satisfying $\text {dist}(y_0)= |y_0-z|=2 r$

4.26 $$\begin{aligned} {[}v-Q_zd^\mu ]_{C^{\beta -1}(\overline{B_r(y_0)})}\le C r^{\mu }, \end{aligned}$$

and

4.27 $$\begin{aligned} \Vert v-Q_zd^\mu \Vert _{L^\infty ({B_r(y_0)})}\le C r^{\mu +\beta -1}, \end{aligned}$$

Then, 

4.28 $$\begin{aligned} \Big \Vert \frac{v}{d^{\mu }}\Big \Vert _{C^{\beta -1}({\overline{\Omega }} \cap B_{1/2})} \le C. \end{aligned}$$

More in general,  if there is an integer $k>\beta -1$ such that $v\in C^k(\Omega \cap B_1)$ and such that for every $\lambda \in \mathbb {N}^n$ with $|\lambda | =k,$

4.29 $$\begin{aligned} \Vert \partial ^\lambda (v-Q_zd^\mu )\Vert _{L^\infty (B_r(y_0))}\le C_k r^{\mu +\beta -1-k}, \end{aligned}$$

then

4.30 $$\begin{aligned} |\partial ^\lambda \big (v/d^{\mu }\big )\big | \le C_kd^{\beta -1-k}\quad \text {in}\ \Omega \cap B_\frac{1}{2}. \end{aligned}$$

### Proof

The proof of the first part of this lemma is essentially contained in the proof of [1, Theorem 1.4]. Let $x_1, x_2\in B_r(y_0)$ and let $\lambda \in \mathbb {N}^n$ be a multi-index with $|\lambda | = \lfloor \beta \rfloor -1.$ Let $Q_z$ as in (4.26), arguing as in the proof of [1, Theorem 1.4], we have the identity

4.31 $$\begin{aligned}  &   \partial ^\lambda (d^{-\mu }u)(x_1)-\partial ^\lambda (d^{-\mu }u)(x_2)\nonumber \\  &   \quad = \sum _{\alpha \le \lambda } [\partial ^\alpha (u-Q_zd^\mu )(x_1) - \partial ^\alpha (u-Q_zd^\mu )(x_2)]\partial ^{\lambda -\alpha }d^{-\mu }(x_1)\nonumber \\  &   \qquad + \partial ^\alpha (u-Q_zd^\mu )(x_2)[\partial ^{\lambda -\alpha }d^{-\mu }(x_1)-\partial ^{\lambda -\alpha }d^{-\mu }(x_2)], \end{aligned}$$

where we have used that $\partial ^\lambda Q_z(x_1) = \partial ^\lambda Q_z(x_2)$ since $Q_z$ has degree $\lfloor \beta -1\rfloor = \lambda .$

We start bounding the terms in the right hand side of (4.31), recalling from Lemma 2.1 that

4.32 $$\begin{aligned} |\partial ^{\lambda -\alpha }d^{-\mu }(x_1)|\le Cr^{|\alpha |-|\lambda |-\mu }, \end{aligned}$$

for any $\alpha \le \lambda .$ On top of this, we can combine (4.32) with the mean value theorem to deduce

4.33 $$\begin{aligned} |\partial ^{\lambda -\alpha }d^{-\mu }(x_1)-\partial ^{\lambda -\alpha }d^{-\mu }(x_2)|\le Cr^{|\alpha |-\mu -\beta }|x_1-x_2|^{\beta -\lfloor \beta \rfloor }. \end{aligned}$$

On the other hand, we can use interpolation [23, Lemma 6.32] to deduce from (4.26) and (4.27) that

4.34 $$\begin{aligned} {[}\partial ^\alpha (v-Q_zd^\mu )]_{C^{\beta - \lfloor \beta \rfloor }(\overline{B_r(y_0)})}\le C r^{\mu -|\alpha |+ \lfloor \beta \rfloor -1} \end{aligned}$$

for any $\alpha \le \lambda .$ In the same vein, we have that

4.35 $$\begin{aligned} \sum _{|\alpha | \le \lfloor \beta \rfloor -1} r^{|\alpha |} \Vert \partial ^{\alpha }(v-Q_zd^\mu )\Vert _{L^\infty (B_r(y_0))}\le C r^{\mu +\beta -1}. \end{aligned}$$

Hence, by combining (4.31), (4.32), (4.34), and (4.35) we deduce

$$\begin{aligned} |\partial ^\lambda (d^{-\mu }u)(x_1)-\partial ^\lambda (d^{-\mu }v)(x_2)|\le C|x_1-x_2|^{\beta -\lfloor \beta \rfloor }, \end{aligned}$$

from where (4.28) follows.

Let us assume now that (4.29) holds for some $k > \lfloor \beta \rfloor -1.$ Let $x\in B_r(y_0)$ and $\lambda \in \mathbb {N}^n$ with $|\lambda | = k.$ Since $\partial ^{\lambda }Q_z=0,$ we can proceed as in (4.31) to get

4.36 $$\begin{aligned} \partial ^\lambda (d^{-\mu }u)(x) = \partial ^\lambda (d^{-\mu }v-Q_z)(x) = \sum _{\alpha \le \lambda } \partial ^\alpha (v-d^{\mu }Q)(x)\, \partial ^{\lambda -\alpha } d^{-\mu }. \end{aligned}$$

Using again [23, Lemma 6.32], we can interpolate (4.27) and (4.29) to deduce

4.37 $$\begin{aligned} \sum _{|\alpha | \le k} r^{|\alpha |} \Vert \partial ^{\alpha }(v-Q_zd^\mu )\Vert _{L^\infty ({B_r(y_0)})}\le C r^{\mu +\beta -1-k}. \end{aligned}$$

Altogether (4.32), (4.36), and (4.37) implies

$$\begin{aligned} |\partial ^\lambda (d^{-\mu }v)(x)|\le C r^{\beta -1-k}\le C d^{\beta -1-k}(x), \end{aligned}$$

where the last inequality follows from the comparability of $d(y_0)$ and *r*. $\square$

### Proof of Theorem 1.2

Let us start by normalizing both sides of (1.9) dividing by $\Vert f\Vert _{C^{k,\alpha }({{\overline{\Omega }}}\cap B_1)} + \Vert v\Vert _{L^\infty (\Omega \cap B_1)}.$ Thus, in virtue of Lemma 4.3, the proof will follow from verifying (4.26). Let $\beta = k+1+\alpha$ and let us invoke Theorem 4.1 if $k=1$ and Theorem 4.2 when $k\ge 2,$ to guarantee the existence of a polynomial $Q_{z} \in {\textbf{P}}_{\lfloor \beta \rfloor -1}$ for each $z \in \partial \Omega \cap B_1$ such that

4.38 $$\begin{aligned} \Big |v(x)-Q_{z}(x)d^{\theta _+}(x)\Big |\le C |x-z|^{{{\theta _+} + \beta -1}}\quad \text{ for } x\in \Omega \cap B_{1}(z). \end{aligned}$$

For any $y_0 \in \Omega \cap B_\frac{1}{2}(z)$ such that $\text {dist}(y_0)= |y_0-z|=2 r$ we define the rescaling

$$\begin{aligned} v_r(x) = r^{{{-\theta _+} - \beta +1}}v(y_0+rx)- r^{1 - \beta }Q_{z}(y_0+rx)b_r(x)^{\theta _+}, \end{aligned}$$

where $b_r(x)= \frac{d(y_0+rx)}{r}.$ Notice that (4.38) implies the bound $\Vert v_r\Vert _{L^\infty (B_1)}\le C.$ On the other hand, the equation for $v_r$ can be written as

4.39 $$\begin{aligned} -\Delta v_r+ a_r(x) v_r= r^{1 - \beta }\Big (f(y_0+rx)b_r(x)^{\theta _+-2}- r^2L_\kappa \Big (Q_{z}(y_0+r\cdot )b_r^{\theta _+}\Big )(x)\Big )\nonumber \\ \end{aligned}$$

with $a_r(x)=\frac{\kappa }{b_r(x)^2}.$ On the other hand, Lemma 2.1 implies that $\frac{1}{C}\le |b_r|\le C$ and $| D^{\lambda } b_r| \le C_{\lambda }$ for $x\in B_1$ and for any multi-index $\lambda .$ So, if $\beta \in (1,2),$ classical gradient estimates yield

4.40 $$\begin{aligned} {[} v_r ]_{C^{\beta -1}(B_{1/2})}\le &   C\Big ( \Vert v_r\Vert _{L^\infty (B_1)}+ \Vert r^{-\beta +1}f(y_0+r \cdot )b_r^{\theta _+-2}\Vert _{L^\infty (B_1)}\Big )\nonumber \\  &   + C r^{{ 3-\beta }}\Vert L_\kappa (Q_{z}(y_0+r\cdot )b_r^{\theta _+})\Vert _{L^\infty (B_1)}. \end{aligned}$$

Moreover, we have from Lemma 2.1 that

4.41 $$\begin{aligned} |L_\kappa (Q_z(y_0+r\cdot )b_r)|\le C b_r(x)^{\beta +\theta _+-3}\le C \end{aligned}$$

and, by the $C^{\beta -1}$ regularity on *f*,  $|f(y_0+rx)|\le Cr^{\beta -1}.$ Thus, by rescaling (4.40), we have

4.42 $$\begin{aligned} r^{-\theta } [ v ]_{C^{\beta -1}(B_{r/2})}\le C. \end{aligned}$$

If $\beta >2,$ since *f* vanishes identically on the boundary, we can write $f d^{\theta _+-2}= f_0d^{\theta _+-1}$ with $f_0 \in C^{\beta -2}({\overline{\Omega }}\cap B_1).$ Additionally, in virtue of Lemma 2.1, we have that

4.43 $$\begin{aligned} L_\kappa (Q_{z}d^{\theta _+})(x)=- g(x)d(x)^{\theta _+-1} \end{aligned}$$

for some $g \in C^{\beta -2}({\overline{\Omega }}\cap B_1).$ Altogether, these observations allow us to rewrite (4.39) as follows

$$\begin{aligned} -\Delta v_r+ a_r(x) v_r= r^{2 - \beta }\Big (f_0(y_0+rx)+g(y_0+rx)\Big )b_r(x)^{\theta _+-1}, \end{aligned}$$

which can be rewritten in the form

4.44 $$\begin{aligned} \frac{-1}{b_r(x)^{\theta _+-1}}\Delta v_r+\frac{a_r(x)}{b_r(x)^{\theta _+-1}} v_r= r^{2 - \beta }\Big (f_0(y_0+rx)+g(y_0+rx)\Big ). \end{aligned}$$

Thus, since $b_r \in C^\beta (B_1)$ and $\frac{1}{C}\le |b_r|\le C,$ we can apply standard interior Schauder estimates to (4.44) (see, e.g., [31]) to deduce

$$\begin{aligned} {[} v_r ]_{C^{\beta -1}(B_{1/2})}\le C\Big ( \Vert v_r\Vert _{L^\infty (B_1)}+ r^{2-\beta } [f(y_0+r \cdot )+g(y_0+r\cdot )]_{C^{\beta -2}(B_1)}\Big ) \end{aligned}$$

After rescaling, we deduce

4.45 $$\begin{aligned} r^{-\theta } [ v ]_{C^{\beta -1}(B_{r/2})}\le C\Big (1+ [f+g]_{C^{\beta -2}(B_r(y_0))}\Big )\le C, \end{aligned}$$

which concludes the proof. $\square$

We finish this section deriving a modified version of Theorem 1.2 that will be applied to $u-c_\gamma d^{\frac{2}{2-\gamma }}$ in the next section.

### Corollary 4.4

Let $\kappa \ge -\frac{1}{4},$ let $\mu =\theta _+$ if $\kappa \ge 0$ and $\mu \in \{\theta _+,\theta _-\}$ when $\kappa <0.$ Let $\Omega \subset \mathbb {R}^n$ be a $C^{\beta }$ domain with $\beta >2,$ $f\in C^{\beta -2}({{\overline{\Omega }}}\cap B_1),$ and let *d* be the regularized distance to $\partial \Omega$ given by Lemma 2.1.

Let $v \in C(\Omega \cap B_1)$ be any solution to

4.46 $$\begin{aligned} -\Delta v + \kappa \,\frac{v}{d^2}=fd^{\mu -1}\quad \text {in }\Omega \cap B_1. \end{aligned}$$

If it satisfies

4.47 $$\begin{aligned} \displaystyle \lim _{x\rightarrow \partial \Omega } \frac{v}{d^{\theta _+}} = 0, \end{aligned}$$

then, $$v\in C^{\beta -2}({{\overline{\Omega }}}\cap B_{1/2})$$ and

$$\begin{aligned} \left\| v/{d^{\mu +1}}\right\| _{C^{\beta -2}({{\overline{\Omega }}} \cap B_{1/2})}\le C\big (\Vert f\Vert _{C^{\beta -2}({{\overline{\Omega }}}\cap B_1)} + \Vert v\Vert _{L^\infty (\Omega \cap B_1)} \big ), \end{aligned}$$

where $C>0$ depends only on $\Omega ,$ *k*,  $\alpha ,$ *n*,  and $\kappa .$

Furthermore, if there is $k>\beta -2$ such that $v\in C^k(\Omega \cap B_1),$ $f\in C^k(\Omega \cap B_1)$ and

4.48 $$\begin{aligned} |\partial ^{\alpha } f| \le C_{\alpha } d^{\beta -2 -|\alpha |}, \quad \text{ in } \Omega \cap B_1, \end{aligned}$$

for every $|\alpha | \in \{\lfloor \beta \rfloor -1,\ldots , k-2\},$ then

4.49 $$\begin{aligned} |\partial ^\lambda \big (v/d^{\mu +1}\big )\big | \le C_kd^{\beta -2-|\lambda |}\quad \text {in}\ \Omega \cap B_\frac{1}{2}. \end{aligned}$$

for every $$|\lambda | \in \{\lfloor \beta \rfloor -1,\ldots , k\}.$$

### Proof

If $\mu = \theta _+,$ we can apply directly Theorem 1.2 to conclude that $v= h d^{\theta _+}$ for some $h \in C^{\beta -1}.$ On top of this, thanks to the boundary condition (4.47), we have that $h|_{\partial \Omega }\equiv 0$ implying that $h/d \in C^{\beta -2},$ proving the result in this case.

If $\mu = \theta _-,$ we exploit the fact that $\theta _+ >\theta _-$ to apply Theorem 4.2 which combined with the boundary condition (4.47) implies that (4.16) holds with $Q_{1,x_0}=0$ for any $x_0.$ The rest of the argument thus follow proceeding as in the proof of Theorem 1.2.

Assuming now $v\in C^k(\Omega \cap B_1)$ and $f\in C^k(\Omega \cap B_1)$ for some $k>\beta -2$ together with the condition (4.48), we notice that as in the proof of Theorem 1.2, we have that for every $z \in \partial \Omega \cap B_1,$ there exists a polynomial $Q_{z} \in {\textbf{P}}_{\lfloor \beta \rfloor -2}$ such that

4.50 $$\begin{aligned} \Big |v(x)-Q_{z}(x)d^{\mu +1}(x)\Big |\le C |x-z|^{{{\mu +1} + \beta -2}}\quad \text{ for } x\in B_{1}(z)\cap \Omega . \end{aligned}$$

So, as in the proof of Theorem 1.2, for any $y_0 \in \Omega \cap B_\frac{1}{2}(z)$ such that $\text {dist}(y_0)= |y_0-z|=2 r$ we define the rescaling

$$\begin{aligned} v_r(x) = r^{{{-(\mu +1)} - \beta +2}}v(y_0+rx)- r^{2 - \beta }Q_{z}(y_0+rx)b_r(x)^{\mu +1}, \end{aligned}$$

with $b_r(x)= \frac{d(y_0+rx)}{r}.$ Since we are assuming $\beta >2,$ we can argue as in the proof of Theorem 1.2 to show that

4.51 $$\begin{aligned} \frac{-1}{b_r(x)^{\mu -1}}\Delta v_r+\frac{a_r(x)}{b_r(x)^{\mu -1}} v_r= r^{2 - \beta }\Big (f_0(y_0+rx)+g(y_0+rx)\Big ), \end{aligned}$$

with $a_r(x)=\frac{\kappa }{b_r(x)^2}$ and where $g \in C^{\beta -2}({\overline{\Omega }}\cap B_1)$ is given by the identity $L_\kappa (Q_{z} d^{\mu }) = -g(z) d^{\mu -1}$ and thanks to (2.9), satisfies

4.52 $$\begin{aligned} |\partial ^{\alpha } g| \le C_{\alpha } d^{\beta -2 -|\alpha |}, \quad \text{ in } \Omega \cap B_1, \end{aligned}$$

for $|\alpha |>\beta -2.$ Thus, by applying interior Schauder estimates to (4.51), we have that for any multi-index $\lambda$ with $|\lambda | \in \{\lfloor \beta \rfloor -1,\ldots , k\}$

$$\begin{aligned} \Vert \partial ^\lambda v_r \Vert _{L^\infty (B_\frac{1}{2})}\le C\Big ( \Vert v_r\Vert _{L^\infty (B_1)}+ r^{2-\beta } \Vert D^{k-2}(f(y_0+r \cdot )+g(y_0+r\cdot ))\Vert _{L^\infty (B_1)}\Big ) \end{aligned}$$

which by (4.48), after rescaling and combining (4.48) with (4.52), implies

$$\begin{aligned}  &   r^{{{-(\mu +1)} - \beta +2+k}}\Vert \partial ^\lambda v \Vert _{L^\infty (B_\frac{r}{2})} \\  &   \quad \le C\Big (1+ r^{k-\beta }\Vert D^{k-2}(f(y_0+r \cdot )+g(y_0+r\cdot ))\Vert _{L^\infty (B_1)}\Big )\le C. \end{aligned}$$

Finally, we can apply Lemma 4.3, which yields (4.49). $\square$

## Higher order free boundary regularity

This section is devoted to the proof of Theorem 1.1. For this, we first show step (a) in (1.11).

### Theorem 5.1

Let $\gamma \in (0,2)$ and let $\Omega \subset \mathbb {R}^n$ be a $C^\beta$ domain, with $\beta >1$ and $\beta \notin {\mathbb {N}}.$ Let $0\le u \in C^{\frac{2}{2-\gamma }}$ be a nontrivial solution of

5.1 $$\begin{aligned} \begin{aligned} \Delta u&= u^{\gamma -1} \quad \text {in}\ \Omega \cap B_1 \\ u&= 0 \qquad \ \text {on}\ \partial \Omega \cap B_1. \end{aligned} \end{aligned}$$

Then,  we have

$$\begin{aligned} u/d^{\frac{2}{2-\gamma }} \in C^{\beta -1}({{\overline{\Omega }}}\cap B_r)\qquad \text {and}\qquad u_i/d^{\frac{\gamma }{2-\gamma }}\in C^{\beta -1}({{\overline{\Omega }}}\cap B_r) \end{aligned}$$

for any $$r<1.$$

### Proof

The proof is by induction on $\lfloor \beta \rfloor$ and it is done through steps.

*Step 1:* We find that $v:= u - c_\gamma d^{\frac{2}{2-\gamma }}$ satisfies

5.2 $$\begin{aligned} L_\kappa v = h d^{\frac{2}{2-\gamma }-2}, \end{aligned}$$

for some function *h* whose regularity will be improved along the proof.

Computing directly, we have that

5.3 $$\begin{aligned} \Delta v = u^{\gamma -1} - \big (c_\gamma d^{\frac{2}{2-\gamma }}\big )^{\gamma -1} + gd^{\frac{2}{2-\gamma }-2}, \end{aligned}$$

where $c_\gamma$ is given by (1.5), and *g* is given by the identity

5.4 $$\begin{aligned} gd^{\frac{2}{2-\gamma }-2} = \Delta \big (c_\gamma d^{\frac{2}{2-\gamma }}\big ) - \big (c_\gamma d^{\frac{2}{2-\gamma }}\big )^{\gamma -1}, \end{aligned}$$

Now, it follows from Taylor’s Theorem (applied to the function $$t^{\gamma -1}-1$$ at $$t=1)$$ that

$$\begin{aligned} p^{\gamma -1}-q^{\gamma -1}= &   (\gamma -1)(p-q)q^{\gamma -2}\\  &   + (p/q-1)^2 q^{\gamma -1} \int _0^1 (\gamma -1)(\gamma -2) \big (1+t p/q\big )^{\gamma -3}(1-t) dt \end{aligned}$$

for all positive numbers $p,q>0.$ Hence, we deduce that

5.5 $$\begin{aligned} u^{\gamma -1} - \big (c_\gamma d^{\frac{2}{2-\gamma }}\big )^{\gamma -1}=\kappa \frac{v}{d^2} + f d^{\frac{2}{2-\gamma }-2} \end{aligned}$$

where $\kappa$ is given by (1.7) and

5.6 $$\begin{aligned} f:= \big (u/d^{\frac{2}{2-\gamma }}-c_\gamma \big )^2 \int _0^1 (\gamma -1)(\gamma -2) \big (c_\gamma +t u/d^{\frac{2}{2-\gamma }}\big )^{\gamma -3}(1-t) dt. \end{aligned}$$

Combining (5.3) and (5.5) we deduce

5.7 $$\begin{aligned} L_\kappa v = f d^{\frac{2}{2-\gamma }-2} + g d^{\frac{2}{2-\gamma }-2}. \end{aligned}$$

Notice that *f* has at least the same regularity up to the boundary as $u/d^{\frac{2}{2-\gamma }}.$

*Step 2:* We prove the base case. Moreover, we show that for any $\beta \in (1,2)$ we have that

5.8 $$\begin{aligned} u/d^{\frac{2}{2-\gamma }} \in C^{\beta -1}\qquad \text {and}\qquad u_i/d^{\frac{\gamma }{2-\gamma }}\in C^{\beta -1} \qquad \text {for all} \end{aligned}$$

for $$i=1,\ldots n.$$

In light of Proposition 2.3, it suffices to show $u_i/d^{\frac{\gamma }{2-\gamma }}\in C^{\beta -1}.$ On the other hand, we claim that

5.9 $$\begin{aligned} |D^1\big (u/d^{\frac{2}{2-\gamma }}\big )\big | \le Cd^{\beta -2}\quad \text {in }\Omega \cap B_r. \end{aligned}$$

for $$r\in (0,1).$$

Indeed, arguing as in Corollary 4.4, given any $z \in \partial \Omega \cap B_1,$ for any $y_0 \in B_\frac{1}{2}\cap \Omega$ such that $\text {dist}(y_0)= |x_0-z|=2 r$ we can consider the rescaling

$$\begin{aligned} v_r(x) = r^{-\beta +1-\frac{2}{2-\gamma }}v(y_0+rx) \end{aligned}$$

which is bounded in $B_1$ in virtue of the regularity of $u/d^\frac{2}{2-\gamma }.$ Hence, we can rewrite (5.7) as

5.10 $$\begin{aligned} \frac{-1}{b_r(x)^{\frac{2}{2-\gamma }-2}}\Delta v_r+\frac{a_r(x)}{b_r(x)^{\frac{2}{2-\gamma }-2}} v_r= r^{2 - \beta }\Big (f(y_0+rx)+g(y_0+rx)\Big ), \end{aligned}$$

with $b_r(x)= \frac{d(y_0+rx)}{r}$ and $a_r(x)=\frac{\kappa }{b_r(x)^2}.$ Let notice now that since $|g| \le \in C d^{\beta -1}$ thanks to Lemma 2.1 and $|f|\le C d^{2(\beta -1)}$ we have that both functions are bounded. Thus, we can use standard gradient estimates to deduce

$$\begin{aligned} \Vert \nabla v_r \Vert _{L^\infty (B_\frac{1}{2})}\le C\Big ( \Vert v_r\Vert _{L^\infty (B_1)}+ r^{2-\beta } \Vert f(y_0+r \cdot )+g(y_0+r\cdot ))\Vert _{L^\infty (B_1)}\Big )\le C \end{aligned}$$

implying

$$\begin{aligned} \Vert \nabla v \Vert _{L^\infty (B_\frac{r}{2})}\le r^{\frac{2}{2-\gamma }+\beta -2}. \end{aligned}$$

From here, we can deduce (5.9) from Lemma 4.3.

With (5.9) at hand, we notice that the elementary identity

$$\begin{aligned} u_i/d^{\frac{\gamma }{2-\gamma }} = d\partial _i\left( u/{d^{\frac{2}{2-\gamma }}}\right) +cu/{d^{\frac{2}{2-\gamma }}},\end{aligned}$$

yields

$$\begin{aligned}u_i/d^{\frac{\gamma }{2-\gamma }} \in C^{\beta -1}({{\overline{\Omega }}}\cap B_r),\end{aligned}$$

completing the base case.

We shall assume from now on $\beta >2$ and, by inductive hypothesis

5.11 $$\begin{aligned} u/d^{\frac{2}{2-\gamma }} \in C^{\beta -2}({{\overline{\Omega }}}\cap B_1). \end{aligned}$$

In particular, from (5.6) we also have that $f \in C^{\beta -2};$ whereas

5.12 $$\begin{aligned} g\in C^{\beta -1}({{\overline{\Omega }}}\cap B_1), \end{aligned}$$

thanks to Lemma 2.1.

*Step 3:* We claim that

5.13 $$\begin{aligned} |D^k\big (u/d^{\frac{2}{2-\gamma }}\big )\big | \le C_kd^{\beta -2-k}\quad \text {in }\Omega \cap B_r, \end{aligned}$$

for all $$k>\beta -2.$$

The proof of this claim will follow from an inductive application of Corollary 4.4. Let us start notice that the base case when $k =\lfloor \beta \rfloor -1$ follows immediately from the boundedness of *f* and *g* arguing as in the previous step, i.e., we have that

$$\begin{aligned} |D^1\big (u/d^{\frac{2}{2-\gamma }}\big )\big | \le Cd^{\beta -3}\quad \text {in }\Omega \cap B_r. \end{aligned}$$

for $$r\in (0,1).$$

Let us assume now that (5.13) holds for $k \le l$ and let is show its validity for $l+1.$ Since *g* is defined by the identity (5.4), Lemma 2.1 implies that

5.14 $$\begin{aligned} |\partial ^{\alpha } g| \le C_{\alpha } d^{\beta -1 -|\alpha |}, \quad \text{ in } \Omega \cap B_1, \end{aligned}$$

for $|\alpha |>\beta -1.$ On the other hand, from (5.6) we have that $f = H\Big ( u/d^\frac{2}{2-\gamma }\Big )$ with $H \in C^\infty$ -bearing in mind also that $u/d^\frac{2}{2-\gamma }$ is uniformly bounded from below. So, the inductive hypotheses implies that for $|\alpha | \in \{ \lfloor \beta \rfloor -1, \ldots , l\}$

5.15 $$\begin{aligned} |\partial ^{\alpha } f| \le C_{\alpha } d^{\beta -2 -|\alpha |}, \quad \text{ in } \Omega \cap B_1. \end{aligned}$$

Hence, (5.14) and (5.15) together with (5.11) allows us to invoke Corollary 4.4 and show that (5.13) holds with $k=l+1,$ completing the inductive step.

*Step 4:* Our next goal is to show that $f \in C^{\beta -1}({{\overline{\Omega }}}\cap B_r)$ for all $r<1.$ We first prove it at all boundary points on $\partial \Omega .$ Namely, for any $z\in \partial \Omega \cap B_r.$ Let us start observing that by inductive hypothesis we have

$$\begin{aligned}u/d^{\frac{2}{2-\gamma }} - c_\gamma = P_z(x) + O(|x-z|^{\beta -2}),\end{aligned}$$

for all $x\in \Omega \cap B_1,$ for some polynomial $P_z$ satisfying $P_z(z)=0,$ and where the (implicit) constants are independent of $z\in \partial \Omega \cap B_r.$ Now, since $P_z(x)=O(|x-z|)$ we obtain

$$\begin{aligned}\left( P_z+O(|x-z|^{\beta -2})\right) ^2 = P_z^2 + O(|x-z|^{\beta -1}),\end{aligned}$$

provided that $\beta >3,$ and therefore

$$\begin{aligned}\big (u/d^{\frac{2}{2-\gamma }} - c_\gamma \big )^2 = P_z^2 + O(|x-z|^{\beta -1}).\end{aligned}$$

When $\beta <3$ we use Proposition 2.3 instead, to get

$$\begin{aligned}u/d^{\frac{2}{2-\gamma }} - c_\gamma = O(|x-z|^{\alpha })\end{aligned}$$

and choosing $2\alpha =\beta -1$ we deduce

$$\begin{aligned}\big (u/d^{\frac{2}{2-\gamma }} - c_\gamma \big )^2 = O(|x-z|^{\beta -1}).\end{aligned}$$

In both cases, we also have that

$$\begin{aligned}\int _0^1 (\gamma -1)(\gamma -2) \big (c_\gamma +t u/d^{\frac{2}{2-\gamma }}\big )^{\gamma -3}(1-t) dt \in C^{\beta -2},\end{aligned}$$

and therefore

$$\begin{aligned}f(x) = Q_z(x) + O(|x-z|^{\beta -1})\end{aligned}$$

for all $x\in \Omega \cap B_1,$ for some polynomial $Q_z$ satisfying $Q_z(z)=0,$ and where the (implicit) constants are independent of $z\in \partial \Omega \cap B_r.$

This means that *f* is pointwise $C^{\beta -1}$ on $\partial \Omega \cap B_r.$ We now want to combine this information with interior regularity estimates, to deduce that $f \in C^{\beta -1}({{\overline{\Omega }}}\cap B_r).$

Indeed, thanks to step 3 and the Leibniz Rule, choosing $k=\lfloor \beta \rfloor ,$ and using that $\big |u/d^{\frac{2}{2-\gamma }}-c_\gamma \big |\le Cd,$ we have

$$\begin{aligned} \begin{aligned} \big |D^k\big (u/d^{\frac{2}{2-\gamma }}-c_\gamma \big )^2\big |&\le C\sum _{i=0}^{k/2} \big |D^i\big (u/d^{\frac{2}{2-\gamma }}-c_\gamma \big )\big |\cdot \big |D^{k-i}\big (u/d^{\frac{2}{2-\gamma }}-c_\gamma \big )\big | \\&\le Cd^{\beta -2-k}d + C\sum _{i=1}^{k/2} \big |D^i\big (u/d^{\frac{2}{2-\gamma }}\big )\big |\cdot \big |D^{k-i}\big (u/d^{\frac{2}{2-\gamma }}\big )\big | \\&\le Cd^{\beta -1-k} + C\sum _{i=1}^{k/2} (1+d^{\beta -2-k+i}) \\&\le Cd^{\beta -1-k} + Cd^{\beta -1-k}. \end{aligned}\end{aligned}$$

Applying again the Leibniz Rule, we find

$$\begin{aligned}\big |D^kf\big |\le Cd^{\beta -1-k}\quad \text {in }\Omega \cap B_r,\end{aligned}$$

and in particular

$$\begin{aligned}f \in C^{\beta -1}({{\overline{\Omega }}}\cap B_r),\end{aligned}$$

as wanted.

*Step 5:* Our next step consists in running a blow-up argument as in the proof of Theorem 4.2 and show the following:

*Claim:* There exists a constant $C>0$ and a radius $\rho >0,$ such that for every $x_0 \in \partial \Omega \cap B_\rho ,$ there exists a polynomial $Q_{x_0} \in {\textbf{P}}_{\lfloor \beta \rfloor -1}$ such that

5.16 $$\begin{aligned} \Big |v(x)-Q_{1,x_0}(x)d^\frac{2}{2-\gamma }(x)\Big |\le C |x-x_0|^{\frac{2}{2-\gamma } + \beta - 1}\quad \text{ for } x\in B_{\rho }(x_0)\cap \Omega . \end{aligned}$$

*Proof of the claim:* This proof follows the same lines of the proof of Theorem 4.2, for this reason we just sketch the common features of the argument, stressing only on the (slight) variations of our reasoning.

Let us assume $x_0=0$ and argue by contradiction assuming a sequence of domains $\Omega _j$ with $\Vert \Omega _j\Vert _{C^{\beta }}\le 1$ and, in consequence, of distance functions $d_j$ such that for any sequence of polynomials $P_{j} \in {\textbf{P}}_{\lfloor \beta \rfloor -1}$

5.17 $$\begin{aligned} \sup _{r >0} \sup _{j\in \mathbb {N}} \frac{1}{r^{\frac{2}{2-\gamma } + \beta -1}} \Big \Vert v_j-P_{j} d_j^\frac{2}{2-\gamma }\Big \Vert _{L^\infty (B_r)}=\infty , \end{aligned}$$

with

5.18 $$\begin{aligned} L_\kappa (v_j)= h_{j}d_j^{\frac{2}{2-\gamma }-2},\quad \text {in } B_2\cap \Omega \end{aligned}$$

with $$\Vert h_{j}\Vert _{C^{\beta -1}( \overline{\Omega _j}\cap B_1)}\le 1$$ for $$j \in \mathbb {N}.$$

We select the least squares polynomial $Q_{j,r} \in {\textbf{P}}_{\lfloor \beta \rfloor -1}$ characterized by the orthogonality condition

5.19 $$\begin{aligned} \int _{B_r} \Big (v_j -Q_{1,j,r} d_j^\frac{2}{2-\gamma }\Big )P_1(x)d_j^\frac{2}{2-\gamma }=0, \end{aligned}$$

for any $P_1 \in {\textbf{P}}_{\lfloor \beta \rfloor -1}$ for $r>0$ and $j\in \mathbb {N}.$

Let us define

$$\begin{aligned} \theta (r) = \sup _{\rho >r }\sup _{j\in \mathbb {N}} \frac{1}{r^{{\frac{2}{2-\gamma } + \beta -1}}} \Big \Vert v_j -Q_{j,r} d_j^\frac{2}{2-\gamma }\Big \Vert _{L^\infty (B_r)}. \end{aligned}$$

Arguing as in Lemma 6.2, we have that $\lim _{r\rightarrow 0^+} \theta (r)=\infty .$ Thus, we can pick a sequence $\{r_j\}_{j\in \mathbb {N}}$ with $r_j\rightarrow 0^+$ as $j\rightarrow \infty$ such that

5.20 $$\begin{aligned} w_{j}(x):= \frac{ v_{j}(r_j x)-Q_{j,r_j}(r_jx)d_j^\frac{2}{2-\gamma }(r_j x)}{r_j^{{\frac{2}{2-\gamma } +\beta -1}}\theta (r_j)}, \end{aligned}$$

satisfies $$ \Vert w_j\Vert _{L^\infty (B_1)} \in [\frac{1}{2},1]$$ for $$j\in \mathbb {N}.$$

Additionally, the same reasoning of Lemma 6.3 provides the growth bound

5.21 $$\begin{aligned} \Vert w_j \Vert _{L^\infty (B_R)}\le C R^{\frac{2}{2-\gamma }+\beta -1}. \end{aligned}$$

One of the main differences with Theorem 4.2 appears when we compute the Laplacian of the auxiliary term $Q_{1,j,r} d_j^\frac{2}{2-\gamma }.$ More precisely, using (2.5) to deduce that

$$\begin{aligned} \Delta d_j^\frac{2}{2-\gamma }(x)= d_j^\frac{2}{2-\gamma }(x)(R_j(x)d_j(x)+c_\gamma ^{\gamma -2}+d_j(x)E(x)), \end{aligned}$$

with $|E(x)|\le C|x|^{\beta -2}$ and $R_j \in {\textbf{P}}_{\lfloor \beta \rfloor -1}$ with $\Vert R_j \Vert _{L^\infty (B_1)}\le C_\beta ,$ we can argue as in Lemma 6.3 to obtain the expansion

5.22 $$\begin{aligned}  &   \frac{1}{\theta (r) r^{\frac{2}{2-\gamma }+\beta -1}}\Delta \Big (Q_{r}(rx)d_j^\frac{2}{2-\gamma }(r x)\Big )\nonumber \\  &   \quad =\frac{1}{\theta (r) r^{\frac{2}{2-\gamma }+\beta -1}}\frac{\kappa r^2}{d_j^2(rx)}\Big (Q_{1,r}(rx)d_j^\frac{2}{2-\gamma }(r x)\Big ) \end{aligned}$$

5.23 $$\begin{aligned}  &   \qquad +P_{r}(x)\Big ( \frac{d_j(rx)}{r}\Big )^{\frac{2}{2-\gamma }-2}+m_*(r,x) \end{aligned}$$

where $P_{r} \in {\textbf{P}}_{M}$ for some $M \in \mathbb {N}$ depending only on $\beta$ and $\kappa$ and where $|m_*(x,r)|\le Cm(r)$ with *m* only depending on $\beta$ and $\gamma$ and such that $m(r)\rightarrow 0$ as $r\rightarrow 0^+.$

By combining (4.18), (4.22), and exploiting the uniform regularity of the functions $h_{j}$ we deduce

5.24 $$\begin{aligned} L_\kappa ( w_j)= &   \Big ( {\tilde{Q}}_{j}(x)+g_{j}(x) \Big )\Big ( \frac{d_j(rx)}{r}\Big )^{\frac{2}{2-\gamma }-2}+h_{j}(x), \end{aligned}$$

where ${\tilde{Q}}_{j} \in {\textbf{P}}_{M }$ for some $M\in \mathbb {N}$ and $\Vert g_{j} \Vert _{L^\infty (B_2\cap \overline{\Omega _j})}+\Vert h_{j} \Vert _{L^\infty (B_2\cap \overline{\Omega _j})}\rightarrow 0$ as $j\rightarrow \infty .$ Additionally, in virtue of Lemma 3.4, the sequence of polynomials ${\tilde{Q}}_{j}$ are uniformly bounded in *j*.

We consider two cases: if $\gamma \ge \frac{2}{3},$ then $\frac{2}{2-\gamma } = \theta _+ +1;$ whereas, if $\gamma < \frac{2}{3},$ we have that $\frac{2}{2-\gamma } = \theta _- +1.$ In both cases, we invoke Corollary 4.4 to obtain uniform boundary estimates that guarantee equicontinuity for $w_j/d_j^{\frac{2}{2-\gamma }}.$ All in all, the solutions $\{w_j\}_{j\in \mathbb {N}}$ to the sequence of problems (4.104.11) are equicontinuous and, therefore, satisfy that $w_j \rightarrow w_0$ locally uniformly. So, thanks to Lemma 3.4, and (4.8) we deduce that

5.25 $$\begin{aligned} {\left\{ \begin{array}{ll} L_\kappa w_0 = Q(x)(x_n)_+^{\frac{2}{2-\gamma }-2}, \quad \{x_n>0\}\\ \Vert w_0\Vert _{L^\infty (B_R\cap \mathbb {R}^n_+)} \le CR^{{\frac{2}{2-\gamma }}+\beta -1} \end{array}\right. } \end{aligned}$$

with $Q \in {\textbf{P}}_{M}$ for some $M\in \mathbb {N}.$ Additionally, thanks to Corollary 4.4 we have that

5.26 $$\begin{aligned} |w_0|\le C x_n^{\frac{2}{2-\gamma }}. \end{aligned}$$

So, since $w_0$ satisfies (5.25) and (5.26), we can apply Lemma 3.5 to conclude that

$$\begin{aligned} w_0= P_1(x)(x_n)_+^\frac{2}{2-\gamma } \end{aligned}$$

with $P_1 \in {\textbf{P}}_{\lfloor \beta \rfloor -1}.$ However, taking limits in the orthogonality condition (5.19) as $j\rightarrow \infty ,$ yields

$$\begin{aligned} \int _{B_1} w_0 Q_1(x)x_n^\frac{2}{2-\gamma }=0, \end{aligned}$$

for all $Q_1 \in {\textbf{P}}_{\lfloor \beta \rfloor -1}.$ Thus, $w_0=0$ contradicting $\Vert w_0\Vert _{L^\infty (B_1)}\ge \frac{1}{2}.$

*Step 6:* We conclude the induction argument.

Once we know that (5.16) holds, it follows arguing as in the proof of Theorem 1.2 that

$$\begin{aligned}u/d^{\frac{2}{2-\gamma }} \in C^{\beta -1}({{\overline{\Omega }}}\cap B_r).\end{aligned}$$

Moreover, exactly as in step 3, we now have

$$\begin{aligned}\big |D^k\big (u/d^{\frac{2}{2-\gamma }}\big )\big | \le Cd^{\beta -1-k}\quad \text {in}\ \Omega \cap B_r,\end{aligned}$$

for all $k>\beta -1,$ which combined with

$$\begin{aligned} u_i/d^{\frac{\gamma }{2-\gamma }} = d\partial _i\left( u/{d^{\frac{2}{2-\gamma }}}\right) +cu/{d^{\frac{2}{2-\gamma }}},\end{aligned}$$

yields

$$\begin{aligned} \big |D^k\big (u_i/d^{\frac{\gamma }{2-\gamma }}\big )\big | \le Cd^{\beta -1-k}\quad \text {in}\ \Omega \cap B_r.\end{aligned}$$

This means that

$$\begin{aligned}u_i/d^{\frac{\gamma }{2-\gamma }} \in C^{\beta -1}({{\overline{\Omega }}}\cap B_r),\end{aligned}$$

and we are done. $\square$

We can finally give the:

### Proof of Theorem 1.1

Assume that $x_0=0$ and that the inner unit normal vector at 0 is $\nu =e_n,$ and let $\Omega =\{u>0\}.$ Since $\Delta u_i = (\gamma -1)u^{\gamma -2} u_i$ in $\Omega$ for all $i=1,...,n,$ then it follows that

$$\begin{aligned}w:=\frac{u_i}{u_n}\end{aligned}$$

solves

$$\begin{aligned}{\textrm{div}}(u_n^2\nabla w) = 0 \quad \text {in}\ \Omega \cap B_1.\end{aligned}$$

Thanks to Proposition, we have that

$$\begin{aligned} u_n^2\asymp d^s \qquad \text {and} \qquad u_n^2/d^s \in C^\alpha (B_{r_\circ }\cap {{\overline{\Omega }}}) \qquad \text {for any}\quad \alpha \in (0,1),\end{aligned}$$

where $$r_\circ >0$$ and

$$\begin{aligned}s:={\frac{2\gamma }{2-\gamma }}>0.\end{aligned}$$

Hence, we can write the PDE for *w* as

5.27 $$\begin{aligned} {\textrm{div}}\Big (d^s a(x)\nabla w \Big ) = 0 \quad \text {in}\ \Omega \cap B_{r_\circ }, \end{aligned}$$

with $a(x)\in C^\alpha (B_{1/2}\cap {{\overline{\Omega }}})$ and $\lambda \le a(x)\le \Lambda$ for some constants $\lambda ,\Lambda >0.$

Let us see now what the boundary condition for *w* on $\partial \Omega$ is. Again by Propositions 2.3, we have that

$$\begin{aligned}w=\frac{u_i}{u_n} \in C^{\alpha }(B_{r_\circ }\cap {{\overline{\Omega }}})\quad \text {for any}\ \alpha \in (0,1),\end{aligned}$$

and combining this with interior estimates, a standard argument yields $|\nabla w|\le Cd^{\alpha -1}$ in $\Omega \cap B_{r_\circ }.$ In particular, taking $\alpha >1-s,$ we deduce that

$$\begin{aligned} \lim _{t\downarrow 0} \frac{w(z+t\nu )-w(z)}{t^{1-s}} = 0 \quad \text {for all}\ z\in \partial \Omega \cap B_{r_\circ }\end{aligned}$$

or, equivalently,

5.28 $$\begin{aligned} \lim _{t\downarrow 0} t^s \,\nabla w(z+t\nu ) \cdot \nu = 0 \quad \text {for all}\ z\in \partial \Omega \cap B_{r_\circ }. \end{aligned}$$

This means that *w* is a weak solution of (5.27) with Neumann-type boundary conditions (5.28), and therefore it follows from [32, Theorem 1.1] that

$$\begin{aligned} w\in C^{1+\alpha }(B_r\cap {{\overline{\Omega }}}) \quad \text {for any}\ r\in (0,r_\circ ).\end{aligned}$$

But then, exactly as in (1.3), we deduce that $\nu \in C^{1,\alpha }$ and $\partial \Omega \in C^{2,\alpha }.$

Now, since $\Omega$ is $C^{2,\alpha },$ we can apply Theorem 5.1 to find that

$$\begin{aligned}u_i/d^{\frac{\gamma }{2-\gamma }} \in C^{1,\alpha }({{\overline{\Omega }}} \cap B_r ),\end{aligned}$$

and in particular

$$\begin{aligned}a(x)= u_n^2/d^s \in C^{1,\alpha }({{\overline{\Omega }}} \cap B_r).\end{aligned}$$

Hence, we can apply again [32, Theorem 1.1] to deduce that

$$\begin{aligned}w\in C^{2+\alpha }({{\overline{\Omega }}} \cap B_r ) \quad \text {for any}\ r\in (0,r_\circ ).\end{aligned}$$

Iterating this procedure, we find that

$$\begin{aligned} \partial \Omega \in C^\infty \qquad \text {and}\qquad u_i/d^{\frac{\gamma }{2-\gamma }} \in C^\infty .\end{aligned}$$

This yields $u/d^{\frac{2}{2-\gamma }} \in C^\infty$ and, in consequence, $u^\frac{2-\gamma }{2}= \Big (u/d^{\frac{2}{2-\gamma }}\Big )^\frac{2-\gamma }{2} d \in C^\infty ,$ and the theorem is proved. $\square$

## Polynomial lemmas

In this section we gather technical lemmas that provide control over the polynomials used in the least squares approximation in our various blow-up arguments. All of these results can be seen as a generalization to those in [1] regarding the control of their least square polynomials, in the sense that we deal with more than one element in the kernel of the limiting linearized operator.

### Lemma 6.1

Let $\kappa \ge - \frac{1}{4}$ and $\theta _\pm$ as in (1.14). Let $k_1, k_2 \in \mathbb {N},$ $\beta >1,$ and let $\Omega$ be such that $\Vert \partial \Omega \cap B_2\Vert _{C^{\beta }}\le 1.$ There exists $\rho >0$ such that for any $r\in (0,\rho )$ and for any $Q_i \in {\textbf{P}}_{k_i}$ for $i=1,2,$ if

6.1 $$\begin{aligned} \Vert Q_1 d^{\theta _+}+Q_2 d^{\theta _- + 1}\Vert _{L^\infty (\partial \Omega \cap B_r )} \le \theta , \end{aligned}$$

where $\theta >0$ and with $Q_2=0$ if either $\theta _- \le 0$ or if $\kappa = - \frac{1}{4},$ then

6.2 $$\begin{aligned} \Vert Q_1\Vert _{L^\infty (\partial \Omega \cap B_{r})}r^{\theta _+}+ \Vert Q_2\Vert _{L^\infty (\partial \Omega \cap B_r)}r^{\theta _-+ 1}\le C\theta , \end{aligned}$$

and

6.3 $$\begin{aligned} \sum _{|\mu |\le k_1}r^{|\mu |+\theta _+} \big |q^{(\mu )}_1\big |+ \sum _{|\mu |\le k_2}r^{|\mu |+\theta _- +1} \big |q^{(\mu )}_2\big | \le C\theta , \end{aligned}$$

with $$C=C(\rho ,n,k_1,k_2, \kappa ).$$

### Proof

Let us notice first that by the equivalence of norms in finite dimensional spaces, there exists a constant $C=C(k_1,k_2,\kappa )$ such that

6.4 $$\begin{aligned} \sum _{|\mu |\le k_1} \big |p^{(\mu )}_1\big |+ \sum _{|\mu |\le k_2}\big |p^{(\mu )}_2\big |\le &   C(\Vert P_1(x) \Vert _{L^\infty (\mathbb {R}^n_+\cap B_1)}+\Vert P_2(x)\Vert _{L^\infty (\mathbb {R}^n_+\cap B_1)})\nonumber \\\le &   C\Big ( \Vert P_1(x) x_n^{\theta _+} \Vert _{L^\infty (\mathbb {R}^n_+\cap B_1)}+\Vert P_2(x)x_n^{\theta _-+1} \Vert _{L^\infty (\mathbb {R}^n_+\cap B_1)}\Big )\nonumber \\\le &   C \Vert P_1(x) x_n^{\theta _+}+P_2(x)x_n^{\theta _-1+1}\Vert _{L^\infty (\mathbb {R}^n_+\cap B_1)}, \end{aligned}$$

where the last inequality uses fundamentally the linear independence of $x_n^{\theta _+}$ and $x_n^{\theta _-+1},$ i.e., $\theta _+-\theta _- \notin \mathbb {N}.$

Let $F_r:= \frac{1}{r} \Omega .$ By compactness, we can deduce from (6.4) and from the $C^{\beta }$ bound in $\partial \Omega \cap B_2,$ we can find $\rho >0$ such that for $r\in (0,\rho )$ and for any $P_i \in {\textbf{P}}_{k_i},$

6.5 $$\begin{aligned} \sum _{|\mu |\le k_1} \big |p^{(\mu )}_1\big |+ \sum _{|\mu |\le k_2} \big |p^{(\mu )}_2\big |\le &   \Big \Vert P_1(x) \Big \Vert _{L^\infty (F_r\cap B_1)}+\Vert P_2(x) \Vert _{L^\infty (F_r \cap B_1)}\nonumber \\\le &   C \Big \Vert P_1(x) \Big (\frac{d(rx)}{r}\Big )^{\theta _+}+P_2(x)\Big (\frac{d(rx)}{r}\Big )^{\theta _-+1} \Big \Vert _{L^\infty (F_r\cap B_1)}.\nonumber \\ \end{aligned}$$

Let $Q_i(x) \in {\textbf{P}}_{k_i}$ for $i=1,2$ and let $r\in (0,r_0).$ Let us consider the polynomials

$$\begin{aligned} P_1(x) =\sum _{ |\mu |\le k_1} r^{|\mu |+\theta _+} q_1^{(\mu )} x^\alpha , \qquad P_2(x) =\sum _{ |\mu |\le k_2} r^{|\mu |+\theta _-+1} q_2^{(\mu )} x^\alpha . \end{aligned}$$

With this choosing of $P_1$ and $P_2$ in (6.5), we deduce, after a change of variables, that

$$\begin{aligned}  &   \sum _{|\mu |\le k_1}r^{|\mu |+\theta _+} \big |q^{(\mu )}_1\big |+ \sum _{|\mu |\le k_2}r^{|\mu |+\theta _-+1} \big |q^{(\mu )}_2\big |\\  &   \quad \le r^{\theta _+} \Big \Vert Q_1 \Big \Vert _{L^\infty ( \Omega \cap B_r)}+r^{\theta _-+1}\Vert Q_2(x) \Vert _{L^\infty ( \Omega \cap B_r)}\\  &   \quad \le C \Big \Vert Q_1 d^{\theta _+}+Q_2d^{{\theta _-+1}} \Big \Vert _{L^\infty ( \Omega \cap B_r)}, \end{aligned}$$

which yields (6.2) and (6.3). $\square$

### Lemma 6.2

Let $\kappa \ge - \frac{1}{4}$ and $\theta _\pm$ as in (1.14). Let $\beta >1$ with $\beta \notin \mathbb {N},$ and let $\Omega$ be such that $\Vert \partial \Omega \cap B_2\Vert _{C^{\beta }}\le 1.$ Let $v \in L^\infty (B_2)$ and let us assume that for some $\rho >0$ and each $r\in (0,\rho )$ there exists $Q_{1,r} \in {\textbf{P}}_{\lfloor \beta \rfloor -1 }$ and $Q_{2,r} \in {\textbf{P}}_{\lfloor \beta +\theta _+-\theta _- \rfloor }-2$ such that

6.6 $$\begin{aligned} \Vert v-Q_{1,r}d^{\theta _+}-Q_{2,r}d^{\theta _-}\Vert _{L^\infty (B_r)} \le c_0 r^{\theta _++\beta -1}, \end{aligned}$$

where *d* is extended by 0 outside of $\Omega \cap B_2,$ and with $Q_{2,r}=0$ if $\theta _- \le 0$ or if $\kappa = -\frac{1}{4}.$

Then there exists $\tilde{Q_1} \in {\textbf{P}}_{\lfloor \beta \rfloor -1 }$ and $\tilde{Q_2} \in {\textbf{P}}_{\lfloor \beta +\theta _+-\theta _- \rfloor }-2$ such that

6.7 $$\begin{aligned} \Vert v-\tilde{Q_1}d^{\theta _+}-\tilde{Q_2}d^{{\theta _-+1}}\Vert _{L^\infty (B_r)} \le Cc_0 r^{\beta +\theta _+-1}, \end{aligned}$$

for $$r \in (0,\rho ).$$

### Proof

Let us consider (6.7) for the values *r* and 2*r*. Subtracting them and then applying the triangular inequality yields

$$\begin{aligned} \Big \Vert (Q_{1,2r}-Q_{1,r}) d^{\theta _+}+ (Q_{2,2r}-Q_{2,r}) d^{\theta _-+1} \Big \Vert _{{L^{\infty }(B_r)}}\le Cc_0r^{\theta _+ +\beta -1}. \end{aligned}$$

Therefore, Lemma 6.1 implies

6.8 $$\begin{aligned} |q^{(\mu _1)}_{i,r}-q^{(\mu _1)}_{i,2r}|\le Cc_0 r^{\beta -1-|\mu _1|}, \end{aligned}$$

with $$|\mu _1|\le \lfloor \beta \rfloor -1;$$ whereas

6.9 $$\begin{aligned} |q^{(\mu _2)}_{2,r}-q^{(\mu _2)}_{i,2r}|\le Cc_0 r^{\beta -2+\theta _+-\theta _--|\mu _2|}, \end{aligned}$$

for $|\mu _2|\le \lfloor \beta +\theta _+-\theta _- \rfloor -2.$ Let us remark that the latter inequality implies the uniform boundedness in *r* for the differences $|q^{(\mu _i)}_{i,r}-q^{(\mu _i)}_{i,2r}|,$ for $i=1,2.$

On the other hand, applying the triangular inequality in (6.6) for $r=1$ and using again Lemma 6.1, we obtain the bound

6.10 $$\begin{aligned} \Vert Q_{1,1}\Vert _{L^\infty (B_1)}+ \Vert Q_{2,1}\Vert _{L^\infty (B_1)}\le Cc_0+\Vert v\Vert _{L^\infty (B_\rho )}. \end{aligned}$$

The latter bound combined with (6.8) and (6.9) implies the convergence of the sequences $\{q^{(\mu )}_{i,2^{-j}}\}_{j\in \mathbb {N}}$ for $i=1,2$ and for each $\mu .$ Denoting the corresponding limits by $q^{(\mu )}_i,$ we deduce, namely for $i=2,$ adding up dyadically and applying the bound that for any $r \in (0,\rho )$

$$\begin{aligned} |q^{(\mu )}_{2}-q^{(\mu )}_{2,r}|\le &   \sum _{j=0}^{\infty } |q^{(\mu )}_{2,2^{-j}r}-q^{(\mu )}_{2,2^{-j-1}r}|\le C c_0\sum _{j=0}^{\infty } (r2^{-j})^{\beta -2+\theta _+-\theta _--|\mu |}\\\le &   C c_0 r^{\beta -2+\theta _+-\theta _--|\mu |} \sum _{j=0}^{\infty } (2^{-j})^{\beta +\theta _+-\theta _--2-|\mu |}\le C c_0 r^{\beta +\theta _+-\theta _--2-|\mu |}. \end{aligned}$$

The same estimate holds, mutatis mutandis, for $i=1.$

Let us define $\tilde{Q_1} = \sum _{|\mu |\le \lfloor \beta \rfloor -1} q_1^{\mu } x^\mu$ and $\tilde{Q_2} = \sum _{|\mu |\le \lfloor \beta +\theta _+-\theta _- \rfloor -2} q_2^{\mu } x^\mu .$ Applying the triangular inequality together with the previous bound (and its analogue for $\tilde{Q_1})$ yields

$$\begin{aligned} \Vert v-\tilde{Q_1}d^{\theta _+}-\tilde{Q_2}d^{{\theta _-+1}}\Vert _{L^\infty (B_r)}\le &   \Vert v-\tilde{Q_{1,r}}d^{\gamma _1}-\tilde{Q_{2,r}}d^{{\gamma _2}}\Vert _{L^\infty (B_r)} \\  &   + \Vert (Q_{1,r}-\tilde{Q_1})d^{\theta _+}\Vert _{L^\infty (B_r)}\\  &   + \Vert (Q_{2,r}-\tilde{Q_2}) d^{\theta _-+1} \Vert _{L^\infty (B_r)}\\\le &   C c_0 r^{\beta +\gamma _1}, \end{aligned}$$

which proves the result. $\square$

### Lemma 6.3

Let $\kappa \ge - \frac{1}{4}$ and $\theta _\pm$ as in (1.14). Let $\beta >1$ with $\beta \notin \mathbb {N},$ and let $\Omega$ be such that $\Vert \partial \Omega \cap B_2\Vert _{C^{\beta }}\le 1.$ Let $v \in L^\infty (B_2)$ and let us assume that for some $\rho >0$ and each $r\in (0,\rho )$ there exists $Q_{1,r} \in {\textbf{P}}_{\lfloor \beta \rfloor -1 }$ and $Q_{2,r} \in {\textbf{P}}_{\lfloor \beta +\theta _+-\theta _- \rfloor -2}$ such that

6.11 $$\begin{aligned} \Vert v-Q_{1,r}d^{\theta _+}-Q_{2,r}d^{\theta _-+1}\Vert _{L^\infty (B_s)}\le \theta (s) r^{\beta -1+\theta _+}, \end{aligned}$$

where $\theta : (0,\rho )\rightarrow (0,\infty )$ is a non-increasing function,  with

6.12 $$\begin{aligned} \lim _{r\rightarrow 0^+} \theta (r) = +\infty , \end{aligned}$$

and with $Q_{2,r}=0$ if $\theta _- \le 0$ or if $\kappa = -\frac{1}{4}.$

Then

1. 6.13 $$\begin{aligned} \lim _{r\rightarrow 0^+} \frac{q_{i,r}^{(\mu )}}{\theta (r)}=0, \end{aligned}$$
  for $$i=1,2.$$
2. For $r\in (0, \rho )$ the functions
  6.14 $$\begin{aligned} w_{r}(x):= \frac{ v(r x)-Q_{1,r}(r x)d^{\theta _+}(r x)-Q_{2,r}(r x)d^{\theta _-+1}(r x)}{r^{{\theta _+ +\beta -1}}\theta (r)}, \end{aligned}$$
  satisfy the uniform bound
  6.15 $$\begin{aligned} \Vert w_r \Vert _{L^\infty (B_R)}\le C R^{\beta -1+\theta _+}, \end{aligned}$$
  for $$R>1.$$
3. Lastly,  if $$\beta >2,$$
  6.16 $$\begin{aligned}  &   \frac{1}{\theta (r) r^{{\theta _+}+\beta -1}}\Delta \Big (Q_{1,r}(rx)d^{\theta _+}(r x)+Q_{2,r}(rx)d^{\theta _-+1}(r x)\Big )\nonumber \\  &   \quad =\frac{1}{\theta (r) r^{{\theta _+}+\beta -1}}\frac{\kappa r^2}{d^2(rx)}Q_{1,r}(rx)d^{\theta _+}(r x) \nonumber \\  &   \qquad + \frac{1}{\theta (r) r^{{\theta _+}+\beta -1}}\frac{\kappa r^2}{d^2(rx)}Q_{2,r}(rx)d^{\theta _-+1}(r x) \nonumber \\  &   \qquad +P_{1,r}(x)\Big ( \frac{d(rx)}{r}\Big )^{\theta _+-1} \nonumber \\  &   \qquad +P_{2,r}(rx) \Big ( \frac{d(rx)}{r}\Big )^{\theta _--1}\nonumber \\  &   \qquad +m_*(r,x) \end{aligned}$$
  where $P_{i,r} \in {\textbf{P}}_{M}$ for $i=1,2$ and $|m_*(x,r)|\le Cm(r)$ such that $m(r)\rightarrow 0$ as $r\rightarrow 0^+.$ Here *m* and *M* only depends on $\beta ,$ $\kappa ,$ and *n*.

### Proof

By subtracting (6.11) at the values *r* and 2*r* and then applying the triangular inequality, we obtain

$$\begin{aligned} \Big \Vert (Q_{1,2r}-Q_{1,r}) d^{\theta _+}+ (Q_{2,2r}-Q_{2,r}) d^{\theta _-+1} \Big \Vert _{{L^{\infty }(B_r)}}\le C\theta (r) r^{\beta +{\gamma _1}} \end{aligned}$$

Therefore, Lemma 6.1 implies

6.17 $$\begin{aligned} \Vert Q_{1,2r}-Q_{1,r} \Vert _{L^{\infty }(B_{2r})}r^{\theta _+}+\Vert Q_{2,2r}-Q_{2,r} \Vert _{L^{\infty }(B_{2r})} r^{\theta _-+1}\le C \theta (r) r^{\beta -1 +\theta _+}\nonumber \\ \end{aligned}$$

Let $i \in \{1,2\}.$ In virtue of (6.17), we deduce

$$\begin{aligned} | q^{(\mu )}_{1,r}-q^{(\mu )}_{1,2r}|\le C\theta (r) r^{\beta -|\mu |-1}, \end{aligned}$$

and

$$\begin{aligned} | q^{(\mu )}_{2,r}-q^{(\mu )}_{2,2r}|\le C\theta (r) r^{\beta -2+\theta _+-\theta _--|\mu |}. \end{aligned}$$

By using the triangular inequality, it follows that for $i=1,$

6.18 $$\begin{aligned} |q^{(\mu )}_{1,r}-q^{(\mu )}_{1,2^Jr}|\le \sum _{l=0}^{J-1} |q^{(\mu )}_{1,2^{l}r}-q^{(\mu )}_{1,2^{l+1}r}|\le C \sum _{l=0}^{J-1} \theta (r2^{l})(r2^l)^{\beta -|\mu |-1}, \end{aligned}$$

where $|\mu |\le \lfloor \beta \rfloor -1.$ Reasoning similarly, we obtain

6.19 $$\begin{aligned} |q^{(\mu )}_{2,r}-q^{(\mu )}_{2,2^Jr}|\le C \sum _{l=0}^{J-1} \theta (r2^{l})(r2^l)^{\beta -2+\theta _+-\theta _--|\mu |}, \end{aligned}$$

where $$|\mu |\le \lfloor \beta +\theta _+-\theta _-\rfloor -2.$$

Let us notice also that $q^{(\mu )}_{i,s}$ is bounded for $s \in [\rho /4,\rho /2]$ and for $i=1,2.$ Indeed, since $v\in L^\infty (B_\rho )$ and $\theta$ is bounded on [*s*/4, *s*/2],  we deduce from (6.11) and Lemma 6.1 that for $s \in [\rho /4,\rho /2]$

$$\begin{aligned} \Vert Q_{1,s} \Vert _{L^{\infty }(B_s)}s^{\theta _+}+\Vert Q_{2,s} \Vert _{L^{\infty }(B_s)} s^{\theta _-+1}\le C_\rho s^{\beta -1 +\theta _+}+\Vert v\Vert _{L^\infty (B_\rho )}. \end{aligned}$$

From here, the boundedness claim readily follows.

On the other hand, if we take *J* such that $2^J r \in [\frac{\rho }{4}, \rho /2),$ we can use the monotonicity of $\theta$ combined with (6.18) to obtain the estimate

$$\begin{aligned} \frac{|q^{(\mu )}_{1,r}-q^{(\mu )}_{1,2^Jr}|}{\theta (r)} \le C \sum _{l=0}^{J-1} \frac{\theta (\rho 2^{l-J-1})}{\theta (r)}(2^{l-J})^{\beta -|\mu |-1} \le C \sum _{l=1}^{J} \frac{\theta (\rho 2^{-i-1})}{\theta (r)}(2^{-i})^{\beta -|\mu |-1}. \end{aligned}$$

So, since, $|\mu | < {\beta -1}$ and (6.13) holds, we deduce from the dominated convergence theorem that the right hand side of the latter inequality goes to zero as $r\rightarrow 0^+.$ By applying an analogous reasoning to (6.19), we obtain (6.13).

Focusing on (6.15), let us notice that the monotonicity of $\theta$ combined with (6.18) and (6.19) yields

6.20 $$\begin{aligned} |q^{(\mu )}_{1,r}-q^{(\mu )}_{1,2^Jr}|\le C \theta (r) r^{\beta -|\mu |-1} \sum _{l=0}^{J-1} (2^l)^{\beta -|\mu |-1}\le C \theta (r)(2^Jr)^{\beta -|\mu |- 1},\qquad \end{aligned}$$

and

6.21 $$\begin{aligned} |q^{(\mu )}_{2,r}-q^{(\mu )}_{2,2^Jr}|\le C \theta (r)(2^Jr)^{\beta -2+\theta _+-\theta _--|\mu |}, \end{aligned}$$

respectively.

It follows that for any $R>1,$

6.22 $$\begin{aligned}  &   \Vert (Q_{1,r }-Q_{1,R r })d^{\theta _+}\Vert _{L^\infty (B_{Rr})}+\Vert (Q_{2,r }-Q_{2,R r })d^{\theta _-+1}\Vert _{L^\infty (B_{Rr})}\nonumber \\  &   \quad \le C \theta (r) (Rr)^{\beta -1+{\theta _+}}. \end{aligned}$$

Hence, using the triangular inequality and combining it with (6.11) and (6.22)

$$\begin{aligned} r^{{\theta _+}+\beta -1}\theta (r) \Vert v \Vert _{L^\infty (B_{rR})}= &   \Vert v-Q_{1,r}d^{\theta _+}-Q_{1,2,r}d^{\theta _-+1}\Vert _{L^\infty (B_{Rr})}\\\le &   \Vert v - Q_{1,r R}d^{\theta _+}-Q_{2,rR}d^{\theta _-+1}\Vert _{L^\infty (B_{Rr})}\\  &   + \Vert (Q_{1,r }-Q_{1,R r })d^{\theta _+}\Vert _{L^\infty (B_{Rr})}\\  &   + \Vert (Q_{2,r }-Q_{2,R r })d^{\theta _-+1}\Vert _{L^\infty (B_{Rr})}\\\le &   C\theta (Rr)(Rr)^{\beta -1+{\theta _+}}+C\theta (r) (Rr)^{\beta -1+{\theta _+}}. \end{aligned}$$

Therefore, by the monotonicity of $\theta ,$ (6.15) follows.

We conclude the proof of the Lemma by showing (6.16). Let $f(x) = Q_{1,r}(x)d^{\theta _+}( x)+Q_{2,r}(x)d^{\theta _-+1}( x),$ computing directly we have that

6.23 $$\begin{aligned} \Delta f(x)= &   Q_{1,r}(x) \Delta (d^{\theta _+}(x))+Q_{2,r}(x)\Delta (d^{\theta _-+1}( x)) \nonumber \\  &   +2 \theta _+ d^{\theta _+-1}\nabla Q_{1,r}(x) \cdot \nabla d(x)+2(\theta _-+1)d^{\theta _-}\nabla Q_{2,r}(x)\cdot \nabla d(x)\nonumber \\  &   + d^{\theta _+}(x) \Delta Q_{1,r}(x)+ d^{\theta _-+1}( x) \Delta Q_{2,r}(x) \end{aligned}$$

Since $\Vert \Omega \cap B_{2}\Vert _{C^{\beta }}\le 1,$ Lemma 2.1 provides the expansion

6.24 $$\begin{aligned} d(x)= P(x)+g(x) = \sum _{|\lambda |\le \lfloor \beta \rfloor } p^{(\lambda )}x^\lambda +g(x), \end{aligned}$$

where $P \in {\textbf{P}}_{\lfloor \beta \rfloor }$ with $\Vert P\Vert _{L^\infty (\Omega \cap B_1)} \le C_\beta ,$ and where $g \in C^{\infty }(\Omega \cap B_2)$ satisfies $|\nabla g| \le C_\beta |x|^{\beta -1}.$ Therefore,

6.25 $$\begin{aligned}  &   \frac{r^2 d^{\theta _+-1}}{\theta (r) r^{\theta _+ +\beta -1}} \nabla Q_{1,r}(rx) \cdot \nabla d(rx)\nonumber \\  &   \quad = \frac{r^2 d^{\theta _+-1}}{\theta (r) r^{\theta _+ +\beta -1}}\left( \nabla Q_{1,r}(rx)\cdot \nabla P(rx) + \nabla Q_{1,r}(x)\cdot \nabla g(rx)\right) \nonumber \\  &   \qquad + R_{1,r}(x)\Big (\frac{d(rx)}{r}\Big )^{\theta _+-1}+ m_1(x,r) \end{aligned}$$

where $R_{1,r} \in {\textbf{P}}_{2\lfloor \beta \rfloor -3}$ and $|m_1(x,r)|\le Cm(r)$ with *m* only depending on $\beta$ and $\kappa$ and such that $m(r)\rightarrow 0$ as $r\rightarrow 0^+.$ Analogously,

6.26 $$\begin{aligned}  &   \frac{r^2 d^{\theta _-}}{\theta (r) r^{\theta _+ +\beta -1}} \nabla Q_{2,r}(rx) \cdot \nabla d(rx)\nonumber \\  &   \quad = \frac{r^2 d^{\theta _-}}{\theta (r) r^{\theta _+ +\beta -1}}\left( \nabla Q_{2,r}(rx)\cdot \nabla P(rx) + \nabla Q_{2,r}(x)\cdot \nabla g(rx)\right) \nonumber \\  &   \qquad + R_{1,r}(x)\Big (\frac{d(rx)}{r}\Big )^{\theta _-}+ m_2(x,r) \end{aligned}$$

where $$R_{2,r} \in {\textbf{P}}_{\lfloor 2\beta +\theta _+-\theta _- \rfloor -4}$$ and $$|m_2(x,r)|\le Cm(r).$$

In a similar vein, using (6.24), we deduce that

6.27 $$\begin{aligned}  &   \frac{r^2}{\theta (r) r^{\theta _+ +\beta -1}}\Big ( d^{\theta _+}(rx) \Delta Q_{1,r}(r x)+ d^{\theta _-+1}(r x) \Delta Q_{2,r}(r x)\Big ) \nonumber \\  &   \quad =m_3(x,r) + R_{3,r}(x) \Big (\frac{d(rx)}{r}\Big )^{\theta _+-1}+R_{4,r}(x) \Big (\frac{d(rx)}{r}\Big )^{\theta _-}, \end{aligned}$$

with $R_{i,r} \in {\textbf{P}}_{M}$ for $i=3,4$ for some $M \in \mathbb {N}$ only depending on $\beta$ and $\kappa$ and where $|m_3(x,r)|\le Cm(r).$

On the other hand, thanks to Lemma 2.1, we also have that

6.28 $$\begin{aligned} \Delta d^{\theta _+}(x) = \frac{\kappa }{d^2(x)} d^{\theta _+} + d^{\theta _+-1} \Big (P_0(x)+g_2(x)\Big ), \end{aligned}$$

with $P_0 \in {\textbf{P}}_{M}$ for some *M* depending on $\beta$ and $|g_2(x)| \le C|x|^{\beta -1}.$ Combining (6.23), (6.25), (6.26), (6.27), (6.28), and (2.4) applied to $d^{\theta _-+1},$ we deduce

$$\begin{aligned} \frac{r^2}{\theta (r) r^{\theta _+ +\beta -1}}\Big ( \Delta f(rx) -\frac{\kappa }{d(rx)^2} f(rx)\Big )= &   P_{1,r}(x) \Big (\frac{d(rx)}{r}\Big )^{\theta _+-1}\\  &   +P_{2,r}(x) \Big (\frac{d(rx)}{r}\Big )^{\theta _-} + m_*(r,x) \end{aligned}$$

with $P_{i,r} \in {\textbf{P}}_{M}$ for $i=1,2$ for some $M \in \mathbb {N}$ only depending on $\beta$ and $\kappa$ and where $|m_*(x,r)|\le Cm(r).$ This proves (6.16). $\square$

## Data Availability

All data needed are contained in the manuscript.

## Footnotes

1 Throughout the paper, we denote $C^\beta =C^{k,\alpha }$ with $k+\alpha =\beta$ and $\alpha \in (0,1],$ $k\in {\mathbb {N}}.$

2 Intuitively, this is because blow-ups of solutions at regular points converge to the function $c_\gamma (x\cdot \nu )_+^\frac{2}{2-\gamma },$ the one dimensional solution of the Alt–Phillips equation.

3 When $m=0,$ we have instead that $w^2$ is $C^{2\alpha }$ at 0.

4 Here, one has to be careful with the case $k=1,$ in which the boundary condition $\partial _\nu w$ must be understood in an appropriate sense.
